# Anomalous Microwave Absorption Hysteresis in Ultrathin MXene Films with Confined Water

**DOI:** 10.1021/acsnano.6c07970

**Published:** 2026-09-24

**Authors:** Changhoon Park, Jamal AlHourani, Yuan Zhang, Jongyoun Kim, James FitzPatrick, Teng Zhang, Gary Friedman, Yury Gogotsi

**Affiliations:** † Materials Science and Engineering Department and A. J. Drexel Nanomaterials Institute, 6527 Drexel University, 3141 Chestnut Street, Philadelphia, Pennsylvania 19104, United States; ‡ Electrical and Computer Engineering Department, Drexel University, 3141 Chestnut Street, Philadelphia, Pennsylvania 19104, United States

**Keywords:** MXenes, intercalation, water confinement, electromagnetic interference shielding

## Abstract

MXenes, a family of two-dimensional (2D) transition-metal carbides, nitrides, and carbonitrides, are promising materials for electromagnetic interference (EMI) shielding due to their electronic conductivity, tunable surface functionalization, and adjustable interlayer spacing. In many practical environments, MXene films host water molecules between the carbide or nitride nanosheets. However, the influence of confined water on their microwave absorption remains unclear. Here, we investigate the role of water confinement in governing microwave absorption in Ti_3_C_2_T_*x*_ MXene films. In situ humidity-dependent measurements reveal that protonated films exhibit a 56% increase in absorption even without measurable changes in interlayer spacing. In contrast, Li^+^-intercalated films, which undergo interlayer swelling upon water uptake, show a 167% increase in absorption. The observed enhancement in microwave absorption upon water uptake is approximately 4 orders of magnitude larger than what would be expected from the increase in bulk water content alone. These changes indicate that water confined within MXene films produces microwave absorption behavior, unlike that of conventional microwave shielding materials where impedance matching is achieved by varying material thickness. The results clarify the role of confined water in EMI absorption in MXenes and indicate that water confinement can serve as a tunable parameter for engineering high-performance EMI shielding materials.

## Graphical abstract

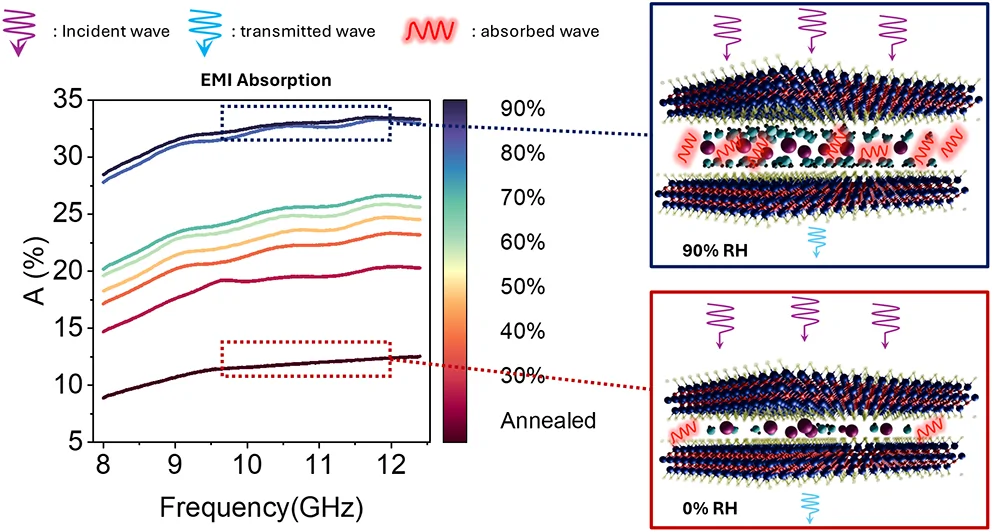

## 1 Introduction

While conductive solids exhibit high shielding effectiveness (SE), they primarily shield by reflection.^1–4^ As their thickness decreases, their sheet impedance increases, leading to increased absorption that reaches a maximum of 50% at typical incidence angles, while reflection and overall shielding effectiveness are reduced. This occurs at very thin metal film thicknesses, often making it difficult to tune the nanostructure needed to make high-quality absorbing microwave screens repeatably. 2D MXenes with the general formula M_*n*+1_X_*n*_T_*x*_, where M is an early transition metal (e.g., Ti, V, Ta, or Nb), X is carbon or nitrogen, T_*x*_ represents surface terminations (−OH, −O, −F, −Cl, etc.), and *n* = 1–4, have been widely explored in EMI shielding applications due to their electronic conductivity, tunable surface functionalization, and adjustable interlayer spacing.^1,5–8^ MXene films with electronically conductive flakes and layered architecture form nanostructures enabling efficient attenuation of microwave radiation^4,9–15^ and strong reflectivity at larger thicknesses. Specifically, increasing the interlayer spacing in Ti_3_C_2_T*_x_* MXene through large-molecule intercalation has been shown to induce strong absorption in the X-band range.^10^ Thermally annealed Ti_3_CNT*_x_* MXene yields EMI shielding effectiveness of 116 dB in a 40 μm-thick film^16^ with relatively high absorption.

MXenes produced by selective etching in acids are hydrophilic and typically contain monolayers of water and intercalated ions after processing and drying in air.^17^ Water can also penetrate the MXene films during storage and use in humid air, imparting distinct electronic properties^18–20^ and potentially altering their microwave properties. Water confined at the nanoscale exhibits unconventional electronic and optical properties distinct from bulk water. Under 2D confinement, water shows high tunability of its out-of-plane and in-plane dielectric constants, depending on the conditions.^21–23^ These confinement-induced changes appear to arise from structural changes and collective polarization of hydrogen-bonded water networks. Such nanoconfined water is ubiquitous in many 2D materials and can markedly alter their intrinsic properties. The degree of water confinement is tunable both by ambient humidity and by the identity of intercalated cations.^24–30^ Notably, the MXene interlayer spacing shows pronounced humidity-dependent hysteresis, which is markedly suppressed when chaotropic cations are present.^24,25,27^ Such confined water is expected to influence optical/microwave behavior through (i) an anomalously large in-plane dielectric constant reported for nanoconfined water^31^ and/or (ii) microwave absorption via dielectric loss intrinsic to water.^32–34^ However, how nanoscale water confinement specifically governs microwave absorption and EMI shielding in MXene films remains unclear.

In this work, we elucidated that water confinement, rather than interlayer spacing alone, is a critical factor governing EMI absorption in MXene films. We performed *in situ* EMI measurements of cation-intercalated Ti_3_C_2_T*_x_* films in a climate chamber to understand EMI absorption in MXene films, which host confined water. We demonstrate that humidity-dependent microwave absorption in Ti_3_C_2_T*_x_* films is driven by intercalant-dependent confined water, which is supported by *in situ* X-ray diffraction (XRD) data. We find that this anomalous absorption in MXene films deviates from classical impedance matching theory. These findings shed light on the role of confined water in light-matter interaction in MXene.

## 2 Results and Discussion

### 2.1 The Evolution of Water Confinement over Humidity Change

To explore the effect of water confinement on EMI shielding, we prepared 100 nm-thick Ti_3_C_2_T_*x*_ MXene films intercalated with cations that differ in hydration behavior. Cation intercalants can be categorized into two groups according to their hydration enthalpy. Kosmotropic cations with high hydration enthalpy—such as Mg^2+^, Na^+^, Li^+^, and Ca^2+^—strongly bind water and readily draw water molecules into the interlayer space.^27,35,36^ In contrast, chaotropic cations—such as K^+^—show limited hydration.^27,35^ H^+^ is a special case. It has very strong hydration, but it bonds to the MXene surface and can be transported by a proton-hopping mechanism without water molecules. When protons (hydronium ions) are present, water can be removed completely, closing the spacing between the MXene nanosheets.^18^ As a result, each ion-intercalated film absorbs a different amount of water when exposed to increasing humidity. Figure 1a illustrates how confined water evolves as humidity rises. At low humidity, water molecules are confined within an interlayer spacing comparable to a monolayer thickness. As humidity increases below a certain threshold, the water content increases while the interlayer spacing remains nearly unchanged. In the case of kosmotropic cations, the interlayer spacing increases sharply to accommodate a bilayer of water, allowing hydrated clusters to form within the interlayer gap once the humidity exceeds a critical threshold.^25,27^ The evolution of water confinement from stage (2) to stage (3) in Figure 1a illustrates the swelling behavior, characterized by a sharp increase in interlayer spacing.

**Figure 1:**
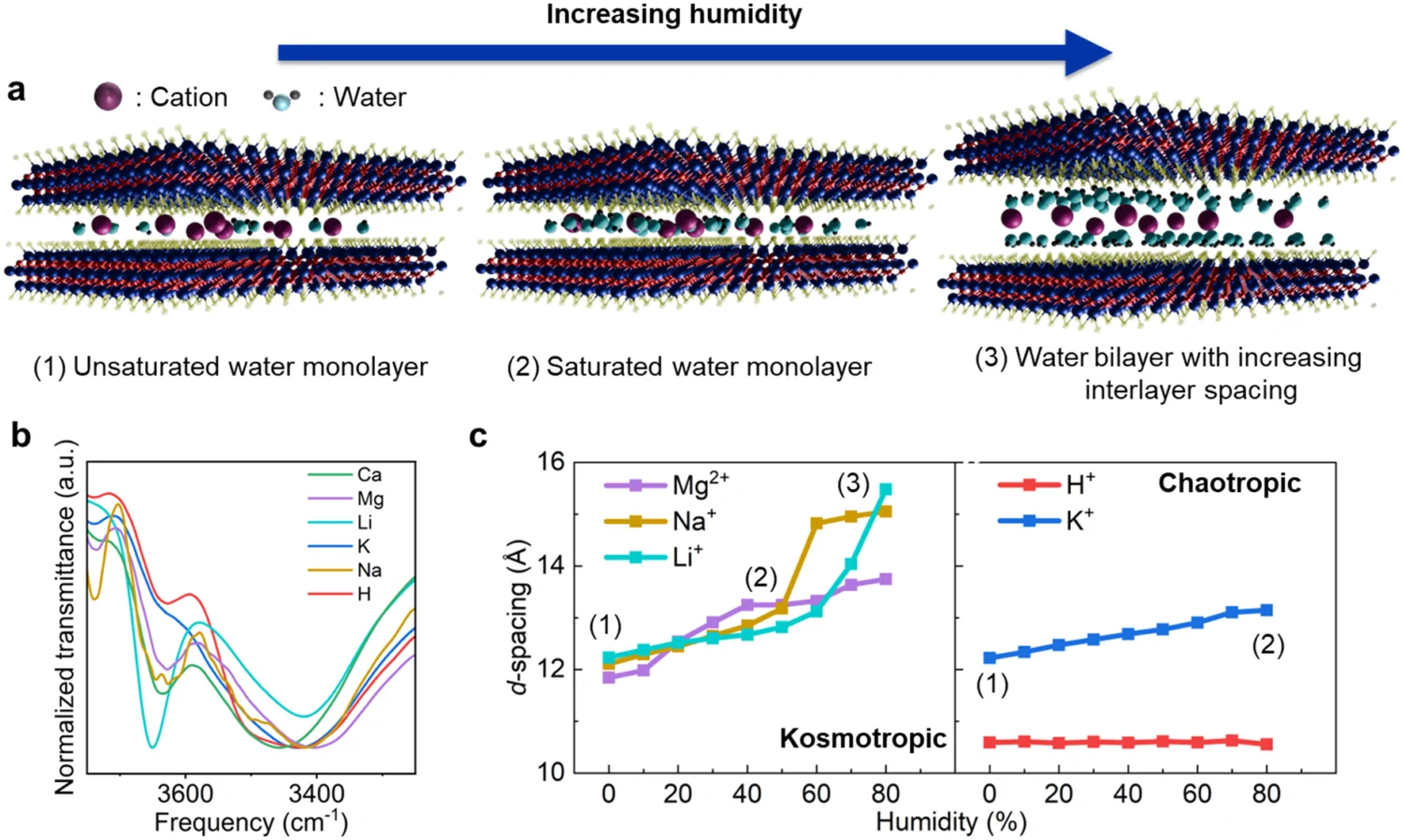
Evolution of water confinement in ion-intercalated Ti_3_C_2_T*_x_* MXene films. (a) Schematic illustration of how confined water changes with humidity. The transition from stage (1) to stage (2) shows an increased amount of water in confinement with minimal variation in interlayer spacing, whereas the transition from stage (2) to stage (3) exhibits interlayer swelling accompanied by substantial water uptake, forming an additional water layer. (b) FTIR spectra of Ca^2+^, Mg^2+^, Li^+^, K^+^, Na^+^, and H^+^-intercalated MXene films at room temperature and humidity. The peaks shown at 3700–3500 cm^–1^, associated with the confined water, are more pronounced in Li^+^-intercalated film compared to the H^+^-intercalated film. (c) Humidity-dependent *d*-spacing measured by XRD for films intercalated with kosmotropic cations (Mg^2+^, Na^+^, and Li^+^) and chaotropic cations (H^+^ and K^+^). Kosmotropic ions show a sharp increase in spacing at a critical humidity, whereas chaotropic ions exhibit only small variations, consistent with monolayer water confinement. The data are taken from the literature.^27^

Figure 1b displays Fourier transform infrared (FTIR) spectra of cation-intercalated films. Regardless of cation identity, all Ti_3_C_2_T_*x*_ MXene films show an absorption peak at 3400 cm^–1^, corresponding to O–H bonding of solvated water.^37^ In contrast, distinct behavior is observed at 3650 cm^–1^ that is assigned to O–H bonding of confined water. While MXene films intercalated with kosmotropic cations (Li^+^, Ca^2+^, Na^+^, and Mg^2+^) reveal strong absorption at 3650 cm^–1^, indicative of significant water confinement, K^+^ and H^+^ ion-exchanged MXene films exhibit much weaker absorption, consistent with limited water confinement. Among kosmotropic cations, Li^+^- intercalated MXene film exhibits relatively high absorption at 3650 cm^–1^ compared to the band associated with solvated water, indicating a large amount of confined water. In addition, X-ray diffraction data (XRD) (Figure S2) show cation-dependent *d*-spacing (see Supporting Information). These observations are in good agreement with previously reported trends, confirming successful cation intercalation in the MXene films.

We have performed a quantitative analysis of different cation-intercalated MXene films and linked the FTIR feature between 3100 and 3700 cm^–1^ to the interlayer spacing changes derived from XRD. Specifically, we demonstrated a correlation between the evolution of this weakly hydrogen-bonded (“confined”) water signature and the variation in the MXene gallery spacing, supporting its assignment beyond a qualitative interpretation.

The humidity-dependent XRD data in Figure 1c quantify these changes in interlayer spacing, as reported in our previous work.^27^ Giving a Ti_3_C_2_T*_x_* monolayer thickness of 9 Å, all ion-exchanged films except the protonated sample show an interlayer spacing of ∼3 Å below 30% relative humidity (RH), consistent with a single water monolayer (∼2.75 Å). In the protonated film (red curve in Figure 1b), H^+^ binds strongly to hydrophilic surface terminations, restricting available space and producing a smaller interlayer spacing of ∼2 Å. Chaotropic cations show only minor changes in spacing (<1 Å) even as humidity increases, indicating that confined water remains limited to a monolayer. In contrast, kosmotropic cations drive a step-like expansion at a critical humidity, corresponding to the transition from stage (1) to stage (2) in Figure 1a.^25,27^ For example, Na^+^- and Li^+^-intercalated films swell beyond 5 Å at 60% RH and 70% RH, respectively, forming a water bilayer. Due to the high hydration enthalpy of Mg^2+^ ions, the hydration-energy barrier suppresses the formation of the first hydration shell, leading to gradual water uptake without abrupt swelling transitions. These results show that water confinement is highly sensitive to humidity and ion identity: kosmotropic intercalants promote swelling and multilayer water formation, whereas chaotropic intercalants restrict water to a monolayer even at high humidity.

### 2.2 Effect of Humidity on EMI Absorption and Electrical Properties

Humidity-induced changes in water confinement directly influence the electromagnetic field interaction with Ti_3_C_2_T_*x*_ MXene films. As water enters the interlayer space, it alters the dielectric environment between adjacent MXene sheets, it increases their polarization, resulting in higher losses during the relaxation processes.^20,31,38^ To examine how these effects modify EMI performance, we measured the scattering parameters (S-parameters) of ion-intercalated Ti_3_C_2_T*_x_* films under controlled humidity conditions inside a climate chamber (see Figure S1 for the experimental setup). The *in situ* experimental setup involved the waveguides to be placed inside the climate chamber during the measurement. The relative humidity was controlled at a constant temperature of 26 °C. Upon reaching each target humidity level, the film was equilibrated for 10 min to ensure sufficient water uptake and stabilization of the interlayer environment prior to S-parameter acquisition. The scattering parameters were then measured and used to calculate the reflection (*R*), transmission (*T*), and absorption (*A*). When these films are exposed to controlled humidity, the absorption increases in a step-like manner. Even below the critical humidity required for structural swelling, all ion-intercalated films absorb water into a monolayer-confined state, demonstrating that confined water alone can increase absorption without requiring changes in interlayer spacing. Then, the films were annealed as a comparison group, exhibiting the smallest water confinement. Figure 2a and b display humidity-dependent absorption spectra. In the annealed state, where interlayer water is minimal and MXene sheets remain closely stacked, the film behaves as a highly conductive reflector, resulting in low absorption (∼10%) (see Figure S4 for humidity-dependent reflectance and transmittance curves).

**Figure 2:**
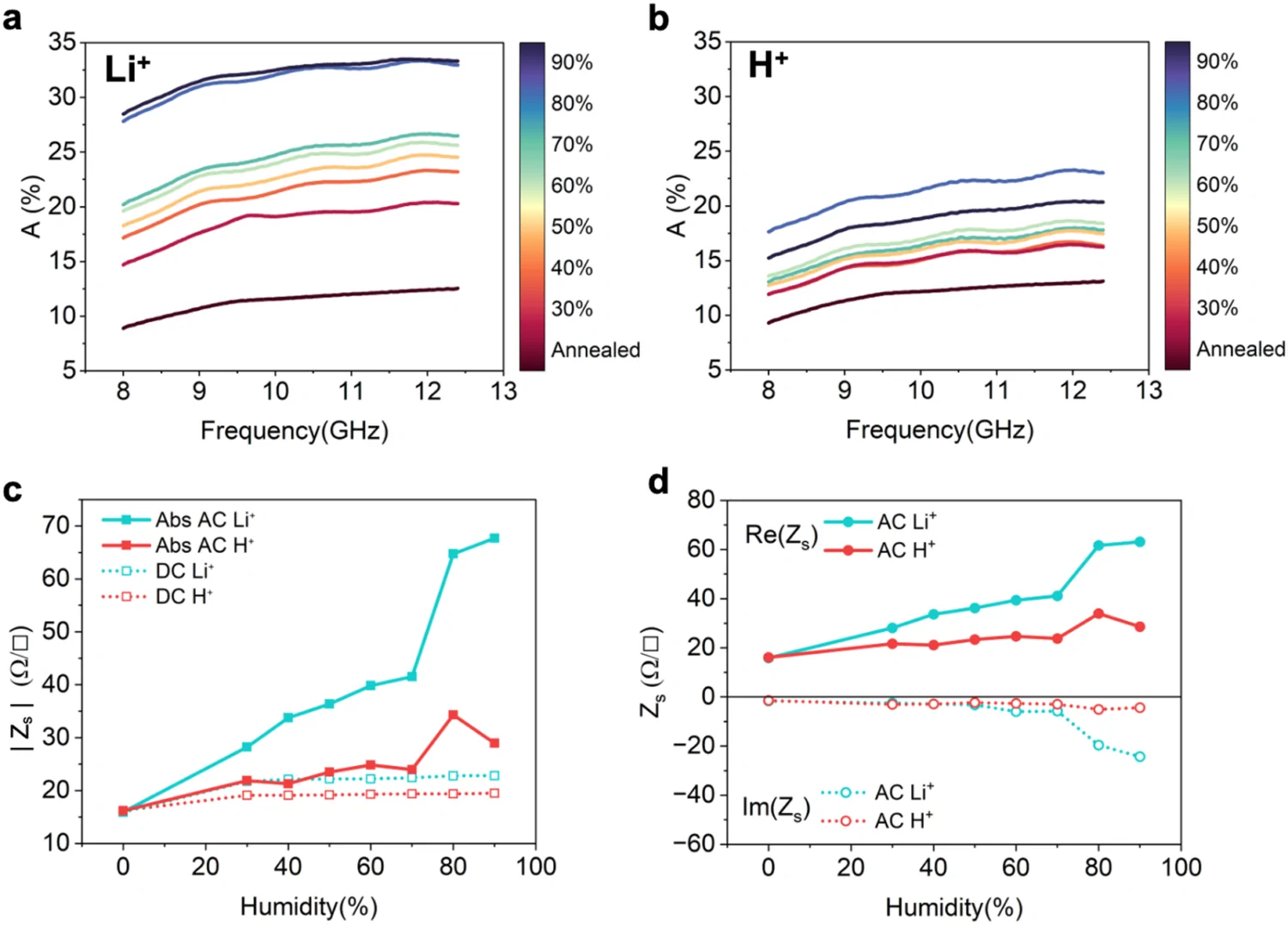
Humidity-induced evolution of microwave absorption and impedance in ion-intercalated Ti_3_C_2_T*_x_* MXene films. (a, b) Humidity-dependent absorption spectrum of (a) Li^+^ intercalated and (b) H^+^ intercalated Ti_3_C_2_T_*x*_ films. Li^+^ films show a sharp, step-like increase in absorption at high humidity due to interlayer swelling, whereas H^+^ films exhibit gradual changes consistent with monolayer water confinement. (c) DC sheet resistance and magnitude of AC sheet impedance as a function of humidity. (d) Real and imaginary components of complex AC impedance in terms of humidity. The upper and bottom panels show real and imaginary parts, respectively.

A key distinction between intercalants emerges at high humidity. Above 70%, Li^+^ and H^+^ ion-exchanged films exhibit markedly different water confinement behaviors. As shown in Figure 2a, the Ti_3_C_2_T_*x*_ film intercalated with Li^+^ undergoes interlayer swelling accompanied by substantial water uptake, leading to a step-like increase in microwave absorption, reaching 167% enhancement relative to its annealed state. In contrast, the protonated Ti_3_C_2_T_*x*_ films display monotonic increases in absorption with rising humidity, but no stepwise increase in absorption is observed in Figure 2b. The absence of a step-like change is likely due to water confinement within approximately a monolayer thickness, leading to minor and continuous changes in interlayer spacing due to an increasing number of water molecules inserted between the MXene nanosheets, as shown in Figure 1b.

The strong influence of water on EMI absorption may arise from its unconventional electromagnetic properties at the nanoscale. Water confined at the nanoscale in graphene exhibits an anomalously large in-plane dielectric constant, reaching values up to 1000, along with ionic conductivity several orders of magnitude higher than that of bulk water.^31^ This is attributable to quasi-2D water with molecules reorienting in-plane and inducing collective polarization. In MXene films, water molecules confined within interlayer gaps comparable to the thickness of monolayer or bilayer water are therefore expected to exhibit a high dielectric constant and strong ionic conduction. These electromagnetic properties of confined water likely influence microwave absorption through two different mechanisms: microwave absorption enhancement in MXene flakes themselves and microwave attenuation by confined water layers.

The humidity-dependent increase in microwave absorption within the MXene flakes themselves can be explained using the classical field solution for a spherical conducting particle of conductivity σ embedded in a dielectric medium, as discussed by Landau and Lifshitz.^39–41^ When the imaginary part σ/ω of the conductor’s relative dielectric constant is much larger than its real part, the polarization established inside such a particle increases with the dielectric constant ϵ_*m*_ of the surrounding medium, yielding the dissipated power density of $P_{inside}=\frac{9\omega^{2}\epsilon_{m}^{2}}{\sigma }{\left|E_{0}\right|}^{2}$ for an external applied electric field of *E*_0_ magnitude. This expression illustrates that the AC power dissipation within the conducting domain can increase quadratically with the surrounding medium dielectric constant ϵ_*m*_. This effect is further amplified in electromagnetically interacting MXene flakes because their polarization increases linearly with their aspect ratio (in-plane size to thickness), as revealed by recent modeling.^42^ Applied to Ti_3_C_2_T_*x*_ films, this suggests that confined water enhances absorption not only by increasing the amount of polarizable material, but also by increasing the effective dielectric constant around the MXene flakes, thereby amplifying polarization currents and Joule dissipation under microwave excitation. Notably, the increase in absorption with humidity is clearly nonlinear, as seen in Figure 3a and b. Although the present measurements do not establish the quadratic dependence on the confined water dielectric constant, the nonlinearity may arise from multiple factors such as the humidity dependence of water uptake as well as changes in the dielectric response of the confined water itself. Thus, while the underlying mechanism may follow from classical electromagnetics, it can be enhanced dramatically by the anomalous increase in the dielectric constant of nanoconfined water. The anomalously high dielectric permittivity of nanoconfined water may also contribute to the modulation of electromagnetic absorption and AC impedance. Based on our previously derived surface integral equation, the ratio of the imaginary to real components of the induced surface charge density scales with the permittivity of the surrounding medium, suggesting that increased permittivity can enhance the out-of-phase charge response associated with AC losses. As the dielectric permittivity of nanoconfined water strongly depends on water layer thickness, changes in both its permittivity and thickness may contribute to the observed absorption and impedance modulation. Such mechanisms are rarely discussed explicitly in the context of layered 2D materials and can be particularly relevant for MXenes.

**Figure 3:**
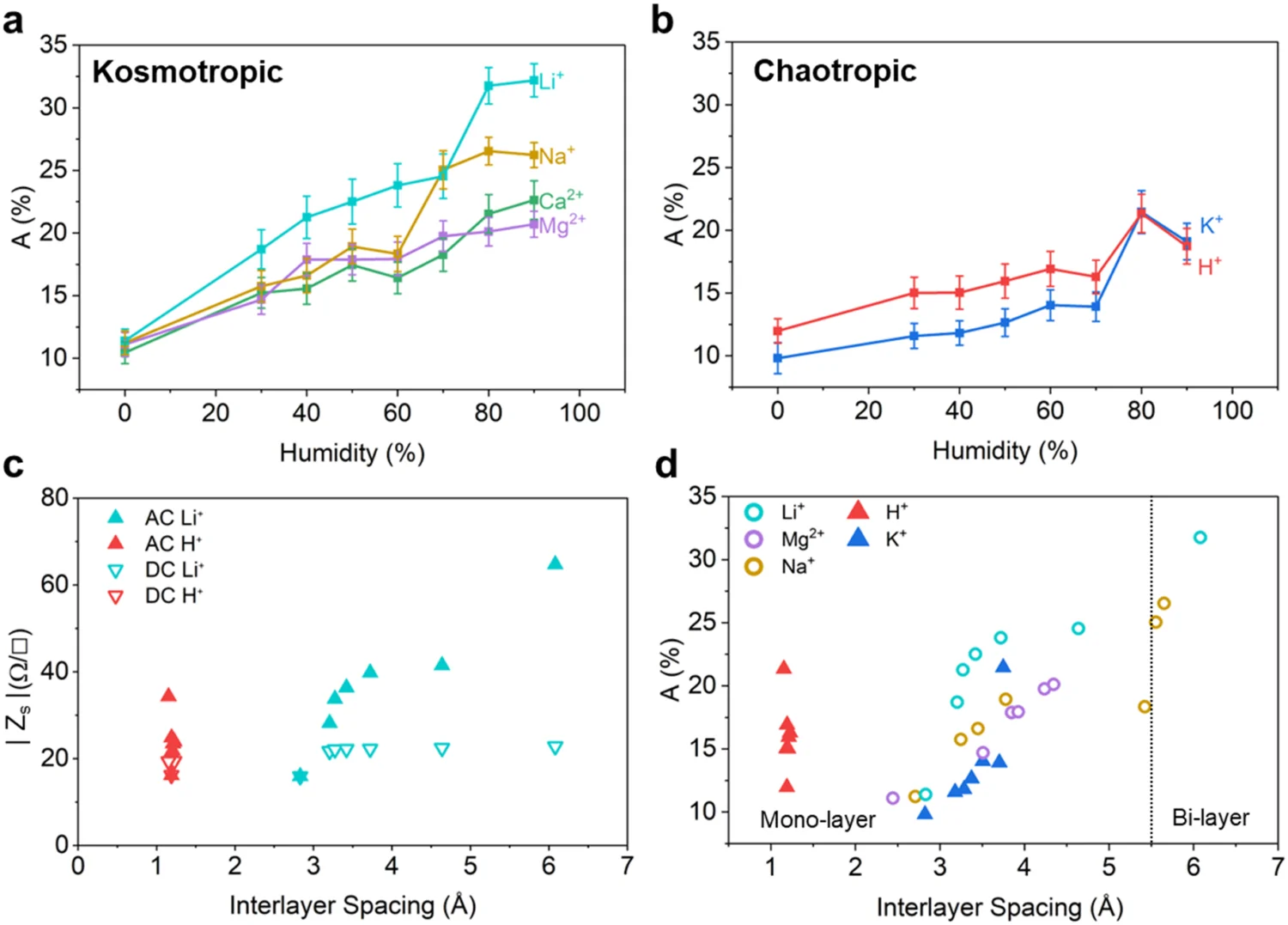
Modulation of microwave absorption and impedance by water confinement in Ti_3_C_2_T*_x_* MXene films. (a, b) Average X-band absorption as a function of humidity for films intercalated with (a) Kosmotropic cations (Li^+^, Na^+^, Ca^2+^, Mg^2+^) and (b) chaotropic cations (H^+^, K^+^). Kosmotropic ions promote greater water uptake and, in some cases, interlayer swelling, resulting in stronger humidity-dependent absorption compared to chaotropic ions. (c) DC sheet impedance and magnitude of AC sheet impedance plotted as a function of interlayer spacing for Li^+^- and H^+^-intercalated films. AC impedance increases with spacing due to reduced interlayer capacitive coupling, whereas DC resistance remains nearly unchanged. (d) Average absorption as a function of interlayer spacing under three water-confinement regimes: monolayer, partially filled monolayer, and bilayer. The bilayer water thickness is assumed to be 5.5 Å, as indicated by the dotted line marking the boundary between the regimes.

For the chaotropic cation-intercalated films, the absorption values exhibit a generally monotonic increase with humidity, though a slight decrease is observed above 80% RH for the K^+^ and H^+^ intercalated samples in Figure 3b. We attribute this minor variation to manual handling of the film during the measurement procedure inside the climate chamber, which can introduce temporary artifacts. Importantly, the overall trend remains robust and reproducible. The reproducibility of the measurements was confirmed by returning the samples to the annealed state after each humidity cycle, which consistently restored the absorption to ∼10%, indicating that the observed changes are fully reversible and dominated by water confinement rather than irreversible sample degradation.

When transitioning from stage (1) to stage (2) in Figure 1a with increasing humidity, the dielectric constant of the interlayer spacing increases with water uptake. This effect accounts for the increase in absorption in MXene films without interlayer swelling. In addition, given recent reports on a substantial increase in ionic conductivity of nanoconfined water,^18,31,43^ it is possible that a large imaginary component of the water permittivity may be responsible for the significant increase in microwave absorption, leading to pronounced microwave absorption.^32–34,44–46^ Beyond microwave properties of water, molecular dynamics (MD) simulations reveal that proton exchange can occur between confined water and hydrophilic surface terminations such as =O and −OH in MXenes, thereby modifying the electronic properties through changes in the electronic state of the surface terminations and underlying metal atoms.^19^

To identify the electrical origin of these behaviors, we obtained direct current (DC) and alternating current (AC) sheet impedances from measurements under different humidity conditions. DC sheet impedance was measured *in situ* using a four-point probe inside the climate chamber, while the complex AC sheet impedance was extracted from the *S*-parameters^47–49^ (See Methods section). Figure 2c displays a comparison between DC and AC impedance as a function of humidity. The annealed films exhibit the lowest sheet impedance in both Li^+^ and H^+^ cases, and the AC and DC values initially agree. After films are exposed to a low-humidity environment, their sheet impedances increase with water uptake, and the AC impedance increases faster with humidity. At any given humidity, AC and DC impedances exhibit distinct humidity-dependent behaviors. Upon water uptake, after an initial increase, the DC sheet resistance remains nearly constant across all humidity levels, indicating that long-range electron percolation through the MXene network is largely preserved. In contrast, the AC sheet impedance increases more rapidly with humidity. In the 30–70% RH range, both films show moderate increases in AC impedance, consistent with insertion of a monolayer of confined water. At 80% RH, Li^+^-intercalated films show a sharp rise in AC impedance, likely reflecting the transition to bilayer-confined water and a corresponding change in dielectric properties. The protonated films, however, exhibit only minor changes due to their limited ability to support multilayer water.

Although humidity-induced structural changes may potentially affect the electromagnetic response of MXene films, the nearly constant sheet resistance over the investigated humidity range in Figure 2c suggests negligible changes in flake alignment and morphology, which would otherwise alter interflake electron transport. However, in the high-frequency regime, intraflake transport is dominant over interflake transport. Even in the absence of significant morphology changes, EM responses of individual flakes are still affected by the dielectric constant of the surrounding medium. We expect that the thickness increase associated with interlayer swelling influences the absorption and AC impedance of MXene films mainly through changes in the dielectric constant of confined water rather than structural changes.

To gain a deeper understanding of the humidity-dependent behavior of the AC impedance, we show the decomposed real and imaginary components in Figure 2d. In the complex impedance plane, a negative imaginary component corresponds to capacitive behavior, signifying enhanced charge storage and dielectric polarization.^31,45,50^ This capacitive response arises from water-filled interlayers acting as nanoscale dielectric gaps between conductive MXene sheets, effectively forming a distributed resistive–capacitive (RC) network that dissipates electromagnetic energy. It is apparent that the increased absorption is also associated with changes in the film’s effective dielectric constant.

The different sensitivities of AC and DC impedance to humidity can be understood from the frequency-dependent effective conductivity, σ­(ω) = σ_DC_ + *iωε*(ω), where the real part represents electronic conduction and the imaginary term arises from the dielectric polarization. In DC, losses are associated with transport, which is dominated by long-range electron percolation through the interconnected MXene flakes.^18,19,51^ Consequently, DC conductivity remains nearly unchanged with humidity. In contrast, AC conductivity measurements capture the imaginary component associated with localized charge accumulation and dipolar oscillation. Because confined water exhibits large permittivity, an increase in interlayer water content leads to substantial changes in AC impedance even when σ_*DC*_ is constant.

A cross-comparison of different cation-intercalated Ti_3_C_2_T*_x_* films elucidates how interlayer structure governs EMI absorption in Figure 3a and b. Li^+^ and Na^+^ intercalated films, which strongly bind water and promote interlayer swelling, produce a step-like increase in absorption once humidity exceeds the swelling threshold. In contrast, Ca^2+^- and Mg^2+^-intercalated films characterized by cations with high hydration enthalpy^18,25^ show a smooth, monotonic absorption increase with humidity. This behavior aligns with their high water uptake capacity, which does not trigger interlayer expansion, consistent with the trends in Figure 1b. Similarly, chaotropic cations such as K^+^ and H^+^ also yield a continuous increase in absorption, but with a much smaller magnitude, reflecting their low hydration enthalpy and correspondingly limited water incorporation without swelling (see Figure S5 for the absorption spectra for each cation).

Figure 3c reveals that the AC sheet impedance is highly correlated to interlayer spacing, while the DC resistance remains essentially unchanged, demonstrating a large effect of confined water on AC losses. Even in the H^+^-intercalated film, where the interlayer spacing remains constant, the AC impedance still changes due to a modulation of its real component. In contrast, Li^+^-intercalated films exhibit increasing AC impedance with swelling interlayer spacing, driven by rises in both the real and imaginary parts of the impedance. This behavior reflects a decrease in effective interlayer capacitance as the spacing between MXene layers increases, weakening capacitive coupling and thereby increasing AC impedance. For other cation-intercalated films, similar trends in DC and AC impedance were observed (Figure S6).

Figure 3d further establishes a clear relationship between EMI absorption and interlayer spacing: films with expanded galleries and multilayer-confined water show substantially higher absorption, driven by stronger dipolar relaxation and polarization.^44,46,52,53^ At low humidity, the interlayer region contains only a monolayer of water, resulting in minimal dielectric loss and, correspondingly, low absorption. As humidity increases, additional water accumulates within this monolayer, amplifying polarization-related losses. At high RH (∼80–90%), Kosmotropic intercalants enable the formation of bilayer-confined water, which significantly enhances dielectric dissipation and yields the highest absorption levels.

The humidity-induced changes in absorption were fully reversible. After completing the humidity cycle, annealing the films to remove intercalated water consistently restored the absorption to its initial annealed state value of ∼10%, confirming that the observed absorption enhancement arises from reversible water confinement rather than irreversible structural degradation or sample damage. This reversibility was observed across all cation-intercalated films, underscoring the robustness of water confinement as a tunable parameter for EMI absorption.

### 2.3 Departure from Classical Impedance-Absorption Relationship in MXenes

Electromagnetic absorption in ultrathin conductive films is often described by the impedance-matching theory, in which maximum absorption occurs when a film’s sheet impedance matches that of the surrounding medium. This framework accurately describes noble metals, graphene, and MXene films measured under constant humidity, as shown in Figure 4 ^10,54–58^ at nongrazing angles of electromagnetic wave incidence.^59^ The sheet impedance of these materials is effectively controlled by their thickness, as the internal architecture remains largely the same. However, Ti_3_C_2_T*_x_* films offer new possibilities, departing sharply from this traditional behavior as humidity and, therefore, water confinement change. Chaotropic K^+^-intercalated films with minimal water confinement show only ∼9.8% absorption, representing a 48% negative deviation from the classical trend. In contrast, Li^+^-intercalated films at 90% RH exhibit a 69% deviation, driven by strong water confinement and interlayer swelling.

**Figure 4:**
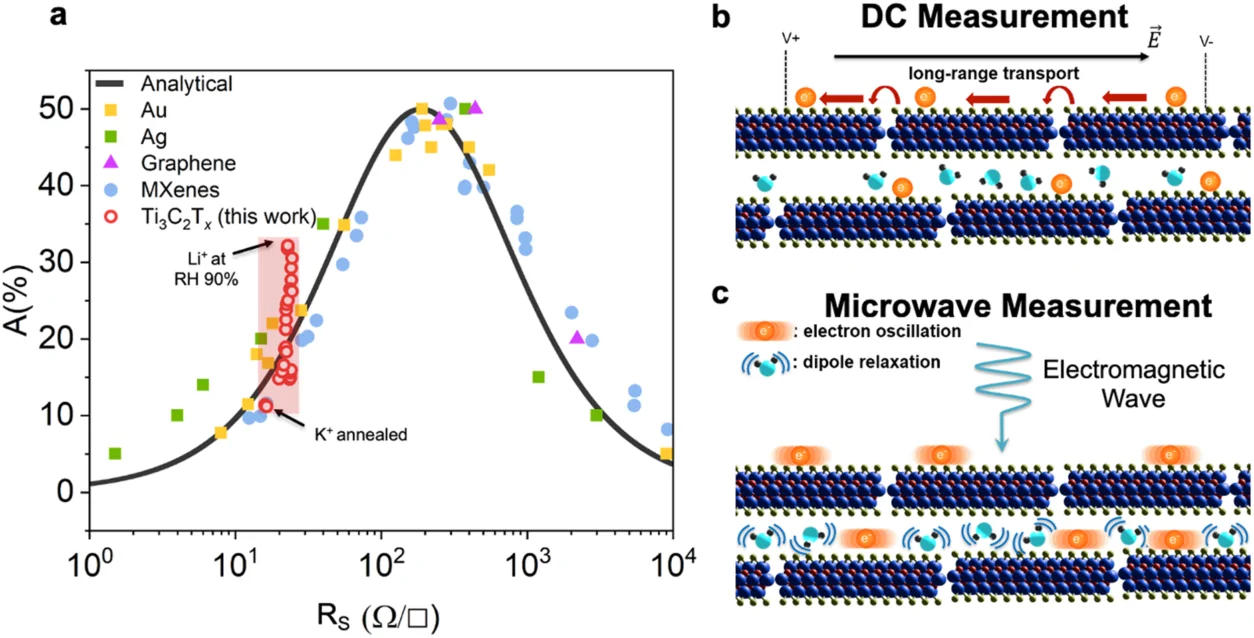
Humidity-induced departure from the classical absorption–impedance relationship in MXene films. (a) Comparison of Ti_3_C_2_T*_x_* MXenes, graphene, and noble metals in the relationship between microwave absorption and DC sheet impedance. MXene films exhibit large deviations from the classical trend when humidity varies, while graphene and metals follow the analytical prediction. (b, c) Schematic illustration of charge transport and microwave response in MXenes under (b) DC and (c) AC excitation. Under DC excitation, long-range electron percolation through stacked MXene flakes dominates and is largely unaffected by humidity. Under AC excitation, confined water introduces strong dielectric polarization and ionic conduction, producing a humidity-sensitive optical response that leads to large deviations from conventional impedance–absorption behavior.

The unconventional deviation arises from the humidity-sensitive nature of AC impedance, which governs EMI absorption, in contrast to the nearly constant DC impedance. Figure 4b and c display different electronic responses of MXene under DC excitation (four-point probe) and microwave excitation (AC). In DC transport, charge carriers propagate through a long-range percolative network formed by continuous MXene flakes, implying that macroscopic conductivity is more strongly governed by alignment of MXene flakes than by humidity-induced changes. Recent studies demonstrate that the dynamic control of flake alignment leads to substantial changes in DC conductivity.^60^ This suggests that water confinement does not significantly disrupt the percolative flake network of MXene film, even with swelling of the interlayer spacing. In contrast, microwave absorption is associated with the microwave response of carriers localized within individual flakes and, possibly, with ionic conduction in confined water.

Our findings highlight the role of water confinement in microwave absorption in 2D materials. In MXenes, the unique architecture composed of conductive layers with hydrophilic surface terminations creates a coupled environment of water and electronic conduction, distinguishing them from other 2D materials. This structural–optical coupling accounts for the high EMI shielding performance observed in ultrathin MXene films: despite their long skin depth, they exhibit strong absorption-dominated shielding compared with thin metallic sheets. In addition, the hydrophilic nature of MXenes enables substantial variation in water confinement, leading to pronounced, humidity-tunable EMI shielding performance. Similar tunable optical responses induced by confined water have also been reported in graphene oxide,^61,62^ suggesting that the mechanisms identified here may be generalizable to other layered 2D materials capable of trapping water between their layers.

## 3 Conclusion

We have demonstrated that confined water plays a critical role in governing the EMI absorption performance of MXene films, extending beyond the mechanisms typically attributed to electrical conductivity and interlayer spacing. Protonated films exhibit a 56% increase in absorption at 90% RH relative to the annealed state, which originates from absorption enhancement in MXene flakes despite negligible changes in interlayer spacing and DC conductivity, implying that the enhanced absorption mainly arises from water confinement. In the MXene film intercalated with a kosmotropic cation, we observed a 167% increase in absorption (Li^+^ case) under the same conditions, attributed to water confinement coupled with interlayer swelling. Compared to bulk water, this absorption enhancement during interlayer swelling is 4 orders of magnitude greater. Given that MXene films exhibit a clear and reversible relationship between *d*-spacing and relative humidity during both increasing and decreasing humidity cycles in in situ XRD measurements, the humidity-induced structural changes are expected to be reversible. Therefore, the corresponding humidity-dependent EMI shielding response is also expected to exhibit good cycling stability, provided that oxidation of the MXene films is prevented. The process is fully reversible, and the absorption decreases after drying the film. These observations deviate from the typical thickness-dominated changes in the film impedance and the resulting absorption relationship, revealing large changes in absorption with minimal variations in sheet resistance. We hypothesize that the strong influence of water confinement on EMI absorption in the MXene film is attributable to the unconventional properties of nanoconfined water, including an anomalously high dielectric constant and a high loss associated with increased polarization of MXene. We believe that the identified mechanism provides new insights into the origins of EMI shielding in MXenes and highlights confined water as a powerful, tunable parameter for modulating microwave response. This understanding offers a framework for designing MXene-based functional devices, including humidity sensors enabled by intercalation of kosmotropic ions and stable EMI shielding films engineered using chaotropic cations.

## 4 Methods

### 4.1 Preparation of MXene Solution and Films

Ti_3_C_2_T*_x_* MXene was synthesized by selectively etching aluminum from the Ti_3_AlC_2_ MAX phase using aqueous HF and HCl at 35 °C for 24 h. The etched material was repeatedly centrifuged and washed with deionized water until the pH reached ∼6, yielding multilayer MXene (ML-MXene) with an accordion-like morphology.^6,17,63–66^ The ML-MXene was delaminated using LiCl (1:1 weight ratio of MAX phase to LiCl) by stirring at 35 °C for 18 h. After removing LiCl by centrifugation and mechanically shaking to promote flake separation, the suspension was centrifuged again. The dark supernatant containing single-layer Ti_3_C_2_T*_x_* was collected, forming a 1 mg mL^–1^ aqueous colloidal solution. The MXene dispersion was spray-coated onto 125 μm-thick polyimide substrates, producing films with a thickness of ∼60 nm. The thickness was characterized using atomic force microscopy (Bruker Icon AFM). Intercalation was done by dipping the MXene films in 0.5 M NaCl, CaCl_2_, MgCl_2_, KCl, HCl, and LiCl solutions for 5 min. After that, the films were rinsed with deionized water to remove the excess salt.

### 4.2 Characterization of Cation-Intercalated MXene Films

XRD measurements were performed using a diffractometer (Rigaku SmartLab, 40 kV, 30 mA) with a Cu Kα X-ray source. The measurements were conducted with a step scan of 0.02° and a step time of 1.25 s. Fourier-transform infrared (FTIR) spectra were collected using an IR Spectrometer (Bruker Invenio R) in attenuated total reflectance mode with a total of 64 scans. The spectral resolution was set to 4 cm^–1^. Baseline correction was performed using a concave rubberband correction (2 iterations, 4 baseline points) while excluding CO_2_ peaks. Spectral smoothing was applied using a 25-point average.

SEM analysis was performed using a Thermo Fisher Apreo 2S Lo Vac scanning electron microscope at an operating voltage of 10 kV and a beam current of 0.20 nA. Images were captured using a secondary electron (SE) detector at magnifications from 120× to 50,000×. All samples were mounted on aluminum stubs with carbon tape.

### 4.3 EMI Measurement and Parameters Extraction

The values of normalized transmission, reflection, and absorption of electromagnetic waves were calculated from the S-parameters measured using a Keysight N5247A vector network analyzer (VNA) and two W*R*-90 rectangular waveguides with the MXene films inserted between the flanges of the waveguides. Before the measurement, thru-reflect-line (TRL) calibration was performed to set the reference nodes at the waveguide flanges.^47,48,67^ Measurements were performed *in situ* inside a climate chamber where the relative humidity was independently varied from 30 to 90% at a constant temperature of 26 °C to monitor the evolution of EMI shielding performance. Before the microwave measurement was done, each film was left in the humidity chamber for 10 min after it reached the target humidity. This was done to ensure sufficient exposure to the programmed humidity despite the ramping rate. After 10 min of exposure to humidity, the measurement was conducted to obtain the *S*-parameters for each case.

The scattering parameters were measured and used to calculate the reflection (*R*), transmission (*T*), and absorption (*A*) using the following equations:

$$\begin{array}{l} R={\left|S_{11}\right|}^{2}\\ T={\left|S_{21}\right|}^{2}\\ A=1‐R‐T \end{array}$$

Importantly, we report the power coefficients (*R, T, A*) rather than the conventional reflection loss (*SE*_R_) and absorption loss (*SE*_A_), as the power coefficients offer a more direct physical interpretation of the fraction of incident electromagnetic power that is reflected, transmitted, or absorbed.^68^

### 4.4 Extraction of AC conductivity

The bare substrates were characterized by measuring *S*_11_ and *S*_21_ to extract the gap parameters of an equivalent Π-network, as shown in Figure S10a:

$$Z_{g3}Y_{\mathrm{gp}}^{2}+2Y_{\mathrm{gp}}=Z_{0}\frac{{\left(1+S_{11}\right)}^{2}-S_{21}^{2}}{2S_{21}}$$

$$Z_{g3}=Z_{0}\frac{{\left(1+S_{11}\right)}^{2}-S_{21}^{2}}{2S_{21}}$$

where $Z_{0}=\omega \mu_{0}/\sqrt{\omega^{2}\mu_{0}\in_{0}‐{\left(\pi /a\right)}^{2}}$ is the TE_10_ mode characteristic impedance inside the waveguide of length *a*, *Z*_*g*3_ is the series impedance of the bare substrate, and *Y*_*gp*_ is the parallel admittance from the bare substrate measurement.^47^ Next, the film-coated samples were measured to obtain *S*_11_^′^ and *S*_21_^′^. The equivalent transmission line model, shown in Figure S10b, was used to calculate the sheet impedance as

$$Z_{s}=\frac{S_{21}^{\prime }Z_{0}}{1-S_{22}-S_{21}-Z_{0}Y_{\mathrm{gp}}\left(1+S_{22}+S_{21}\right)}$$

## Supplementary Material



## Notes

Additional experimental details and characterization data, including the in situ humidity-dependent EMI measurement setup; X-ray diffraction (XRD) and scanning electron microscopy (SEM) characterization of cation-intercalated Ti_3_C_2_T*_x_* films; frequency-dependent reflection, transmission, absorption, and total shielding effectiveness data under varying humidity; DC and AC sheet-impedance measurements; atomic force microscopy (AFM) characterization; the equivalent transmission-line model; and inductively coupled plasma optical emission spectrometry (ICP-OES) methodology and elemental analysis of ion-exchanged films (PDF)

## Abbreviations

2D: two-dimensional
EMI: electromagnetic interference shielding
SE: shielding efficiency
XRD: X-ray diffraction
FTIR: Fourier transform infrared spectroscopy
RH: relative humidity
S-parameters: scattering parameters
MD: molecular dynamics
DC: direct current
AC: alternating current
RC network: resistor-capacitor circuit
ML-MXene: multilayer MXene
VNA: vector network analyzer
TRL: thru-reflect-line calibration
R: reflection
T: transmission
A: absorption
SE_R_: reflection shielding efficiency
SE_A_: absorption shielding efficiency

## References

[1] Iqbal A., Sambyal P., Koo C. M. (2020). 2D MXenes for Electromagnetic Shielding:
A Review. Adv. Funct. Mater..

[2] Celozzi, S. ; Araneo, R. ; Lovat, G. Electromagnetic Shielding. In Encyclopedia of RF and Microwave Engineering; Wiley, 2024; pp 1–22.

[3] Han J.-H., Park J., Kim M., Lee S., Heo J. M., Jin Y. H., Chae Y., Han J., Wang J., Seok S.-H., Sim Y., Byun G., Lee G.-D., Choi E., Kwon S.-Y. (2025). Ultrahigh Conductive
MXene Films
for Broadband Electromagnetic Interference Shielding. Adv. Mater..

[4] Rakhmanov R., Shuck C. E., Al Hourani J., Ippolito S., Gogotsi Y., Friedman G. (2023). Ultrathin MXene Film Interaction with Electromagnetic
Radiation in the Microwave Range. Appl. Phys.
Lett..

[5] Shahzad F., Alhabeb M., Hatter C. B., Anasori B., Hong S. M., Koo C. M., Gogotsi Y. (2016). Electromagnetic
Interference Shielding
with 2D Transition Metal Carbides (MXenes). Science.

[6] Shuck C. E., Sarycheva A., Anayee M., Levitt A., Zhu Y., Uzun S., Balitskiy V., Zahorodna V., Gogotsi O., Gogotsi Y. (2020). Scalable Synthesis of Ti_3_C_2_T*
_x_
* MXene. Adv. Eng. Mater..

[7] Zhang Y., Ruan K., Zhou K., Gu J. (2023). Controlled Distributed
Ti_3_C_2_T_
*x*
_ Hollow Microspheres
on Thermally Conductive Polyimide Composite Films for Excellent Electromagnetic
Interference Shielding. Adv. Mater..

[8] Naguib M., Kurtoglu M., Presser V., Lu J., Niu J., Heon M., Hultman L., Gogotsi Y., Barsoum M. W. (2011). Two-Dimensional
Nanocrystals Produced by Exfoliation of Ti_3_AlC_2_. Adv. Mater..

[9] Iqbal A., Hassan T., Naqvi S. M., Gogotsi Y., Koo C. M. (2024). MXenes
for Multispectral Electromagnetic Shielding. Nature Reviews Electrical Engineering.

[10] Rakhmanov R., Ippolito S., Downes M., Inman A., AlHourani J., Fitzpatrick J., Gogotsi Y., Friedman G. (2025). Influence of MXene
Interlayer Spacing on the Interaction with Microwave Radiation. Adv. Funct. Mater..

[11] Liu J., Zhang H.-B., Sun R., Liu Y., Liu Z., Zhou A., Yu Z.-Z. (2017). Hydrophobic, Flexible, and Lightweight
MXene Foams for High-Performance Electromagnetic-Interference Shielding. Adv. Mater..

[12] Zhu Y., Liu J., Guo T., Wang J. J., Tang X., Nicolosi V. (2021). Multifunctional
Ti_3_C_2_T_
*x*
_ MXene Composite
Hydrogels with Strain Sensitivity toward Absorption-Dominated Electromagnetic-Interference
Shielding. ACS Nano.

[13] Yun T., Kim H., Iqbal A., Cho Y. S., Lee G. S., Kim M.-K., Kim S. J., Kim D., Gogotsi Y., Kim S. O., Koo C. M. (2020). Electromagnetic
Shielding of Monolayer MXene Assemblies. Adv.
Mater..

[14] Lee G. S., Yoon Y. H., Iqbal A., Kwon J., Yun T., Sasikala S. P., Hassan T., Kim J. G., Kim J. T., Lee C. W., Kim M.-K., Koo C. M., Kim S. O. (2023). Maximized
Internal Scattering in Heterostack Ti_3_C_2_T_
*x*
_MXene/Graphene Oxide Film for Effective Electromagnetic
Interference Shielding. 2D Mater..

[15] Lee Y., Yoon Y. H., Lee G. S., Nam K. S., Hassan T., Kim D.-Y., Jo K., Hong J., Koo C. M., Kim S. O., Park S. (2026). Scalable Production
of Tailor-Designed
MXene–Hydrogel Core–Shell Fibers via Dual Stress-Guided
Alignment during Thermal Drawing. ACS Nano.

[16] Iqbal A., Shahzad F., Hantanasirisakul K., Kim M.-K., Kwon J., Hong J., Kim H., Kim D., Gogotsi Y., Koo C. M. (2020). Anomalous Absorption of Electromagnetic Waves by 2D
Transition Metal Carbonitride Ti_3_CNT_
*x*
_ (MXene). Science.

[17] VahidMohammadi A., Rosen J., Gogotsi Y. (2021). The World
of Two-Dimensional Carbides
and Nitrides (MXenes). Science.

[18] Zhang T., Mazzio K. A., Wang R. J., Lounasvuori M., Al-Temimy A., Amargianou F., Mawass M.-A., Kronast F., Többens D. M., Lips K., Petit T., Gogotsi Y. (2025). Conductivity
Hysteresis in MXene Driven by Structural Dynamics of Nanoconfined
Water. Nat. Commun..

[19] Khanal R., Irle S. (2023). Effect of Surface Functional
Groups on MXene Conductivity. J. Chem. Phys..

[20] Jalali H., Khoeini F., Peeters F. M., Neek-Amal M. (2021). Hydration
Effects and Negative Dielectric Constant of Nano-Confined Water between
Cation Intercalated MXenes. Nanoscale.

[21] Fumagalli L., Esfandiar A., Fabregas R., Hu S., Ares P., Janardanan A., Yang Q., Radha B., Taniguchi T., Watanabe K., Gomila G., Novoselov K. S., Geim A. K. (2018). Anomalously Low Dielectric Constant of Confined Water. Science.

[22] Wang Y., Tang F., Yu X., Chiang K.-Y., Yu C.-C., Ohto T., Chen Y., Nagata Y., Bonn M. (2025). Interfaces
Govern the Structure of Angstrom-Scale Confined Water Solutions. Nat. Commun..

[23] Han S., Choi M. Y., Kumar P., Stanley H. E. (2010). Phase Transitions
in Confined Water Nanofilms. Nat. Phys..

[24] Gao Q., Sun W., Ilani-Kashkouli P., Tselev A., Kent P. R. C., Kabengi N., Naguib M., Alhabeb M., Tsai W.-Y., Baddorf A. P., Huang J., Jesse S., Gogotsi Y., Balke N. (2020). Tracking Ion Intercalation
into Layered Ti_3_C_2_ MXene Films across Length
Scales. Energy Environ.
Sci..

[25] Ghidiu M., Halim J., Kota S., Bish D., Gogotsi Y., Barsoum M. W. (2016). Ion-Exchange and Cation Solvation Reactions in Ti_3_C_2_ MXene. Chem. Mater..

[26] Kim J. E., Han T. H., Lee S., Kim J. Y., Ahn C. W., Yun J. M., Kim S. (2011). Graphene Oxide
Liquid Crystals. Angew. Chem., Int. Ed..

[27] Lounasvuori M., Zhang T., Gogotsi Y., Petit T. (2024). Tuning the Microenvironment
of Water Confined in Ti_3_C_2_T_
*x*
_ MXene by Cation Intercalation. J. Phys.
Chem. C.

[28] Lee D. H., Lee W. J., Lee W. J. (2011). Theory,
Synthesis, and
Oxygen Reduction Catalysis of Fe-Porphyrin-Like Carbon Nanotube. Phys. Rev. Lett..

[29] Kim H., Ha Y. G., Kang D.-G., Osuji C. O., Kim D. S., Lee C. (2026). High-Concentration
Mesogen-Assisted Exfoliation of Low-Dimensional
Nanomaterials for Achieving Ultralow-Temperature Actuations of Liquid
Crystal Elastomers. Adv. Funct. Mater..

[30] Lee G. S., Kim J. G., Kim J. T., Lee C. W., Cha S., Choi G. B., Lim J., Padmajan Sasikala S., Kim S. O. (2024). 2D Materials Beyond Post-AI Era:
Smart Fibers, Soft
Robotics, and Single Atom Catalysts. Adv. Mater..

[31] Wang R., Souilamas M., Esfandiar A., Fabregas R., Benaglia S., Nevison-Andrews H., Yang Q., Normansell J., Ares P., Ferrari G., Principi A., Geim A. K., Fumagalli L. (2025). In-Plane Dielectric
Constant and Conductivity of Confined
Water. Nature.

[32] Ellison W. J. (2007). Permittivity
of Pure Water, at Standard Atmospheric Pressure, over the Frequency
Range – 25 THz and the Temperature Range – 100°C. J. Phys. Chem. Ref. Data.

[33] Hong J., Kwon J., Iqbal A., Kim D., Kwon T., Sambyal P., Hong S. M., Yoon H. G., Kim M.-K., Koo C. M. (2022). Electromagnetic Shielding of Optically-Transparent
and Electrically-Insulating Ionic Solutions. Chem. Eng. J..

[34] Liebe H. J., Hufford G. A., Manabe T. (1991). A Model for the Complex
Permittivity
of Water at Frequencies below 1 THz. Int. J.
Infrared Millimeter Waves.

[35] Smith D. W. (1977). Ionic Hydration
Enthalpies. J. Chem. Educ..

[36] Shpigel N., Levi M. D., Sigalov S., Mathis T. S., Gogotsi Y., Aurbach D. (2018). Direct Assessment of
Nanoconfined Water in 2D Ti_3_C_2_ Electrode Interspaces
by a Surface Acoustic
Technique. J. Am. Chem. Soc..

[37] Parker T., Zhang Y., Shevchuk K., Zhang T., Khokhar V., Kim Y.-H., Kadagishvili G., Bugallo D., Tanwar M., Davis B., Kim J., Fakhraai Z., Hu Y.-J., Jiang D., Talapin D. V., Gogotsi Y. (2025). In Situ Raman and Fourier
Transform Infrared Spectroscopy Studies of MXene–Electrolyte
Interfaces. ACS Nano.

[38] Sugahara A., Ando Y., Kajiyama S., Yazawa K., Gotoh K., Otani M., Okubo M., Yamada A. (2019). Negative Dielectric
Constant of Water Confined in Nanosheets. Nat.
Commun..

[39] Bohren, C. F. ; Huffman, D. R. Absorption and Scattering by a Sphere. In Absorption and Scattering of Light by Small Particles; Wiley, 1998; pp 82–129.

[40] Landau, L. D. ; Lifshitz, E. M. ; Skyes, J. B. ; Bell, J. S. ; Kearsley, M. J. ; Pitaevskii, L. P. Electrostatics of Dielectrics. In Electrodynamics of Continuous Media; Elsevier, 1984; Vol. 8, pp 34–85.

[41] Landau, L. D. ; Lifshitz, E. M. ; Skyes, J. B. ; Bell, J. S. ; Kearsley, M. J. ; Pitaevskii, L. P. Electrostatics of Conductors. In Electrodynamics of Continuous Media; Elsevier, 1984; Vol. 8, pp 1–33.

[42] Ghosh R., Gogotsi Y., Friedman G. (2026). Numerical
Simulation of Effective
Dielectric Constant of MXene Films. J. Appl.
Phys..

[43] Milton K. L., Shluger A. (2025). Impact of Confined
Water on the Electronic Structure
of the SiO_2_ and WS_2_ Interface. ACS Appl. Mater. Interfaces.

[44] Liu J., Duan C., Mei W. N., Smith R. W., Hardy J. R. (2005). Dielectric
Properties and Maxwell-Wagner Relaxation of Compounds ACu_3_Ti_4_O_12_ (A = Ca,Bi_2/3_,Y_2/3_,La_2/3_). J. Appl. Phys..

[45] Pething, R. Dielectric Polarization. In Dielectrophoresis; Wiley, 2017; pp 145–166.

[46] Prodromakis T., Papavassiliou C. (2009). Engineering
the Maxwell–Wagner Polarization
Effect. Appl. Surf. Sci..

[47] Wang X.-C., Díaz-Rubio A., Tretyakov S. A. (2017). An Accurate Method for Measuring
the Sheet Impedance of Thin Conductive Films at Microwave and Millimeter-Wave
Frequencies. IEEE Trans. Microwave Theory Tech..

[48] Costa F., Borgese M., Degiorgi M., Monorchio A. (2017). Electromagnetic
Characterisation of Materials by Using Transmission/Reflection (T/R)
Devices. Electronics.

[49] Costa F. (2013). Surface Impedance
Measurement of Resistive Coatings at Microwave Frequencies. IEEE Trans. Instrum. Meas..

[50] Gonella G., Backus E. H. G., Nagata Y., Bonthuis D. J., Loche P., Schlaich A., Netz R. R., Kühnle A., McCrum I. T., Koper M. T. M., Wolf M., Winter B., Meijer G., Campen R. K., Bonn M. (2021). Water at Charged
Interfaces. Nat. Rev. Chem..

[51] Helal M. I., Sinopoli A., Gladich I., Tong Y., Alfahel R., Gomez T., Mahmoud K. A. (2024). Understanding
the Swelling Behavior
of Ti_3_C_2_T_
*x*
_ MXene
Membranes in Aqueous Media. J. Mater. Chem.
A.

[52] Liu L. C., Liang J. C., Chen K. W., Chu K. R. (2023). Effects of Polarization-Charge
Shielding and Electromagnetic Resonances on Water Behavior under Microwave
Heating. Phys. Plasmas.

[53] Sikiru S., Yahya N., Soleimani H., Ali A. M., Afeez Y. (2020). Impact of
Ionic-Electromagnetic Field Interaction on Maxwell-Wagner Polarization
in Porous Medium. J. Mol. Liq..

[54] Mattiucci N., Bloemer M. J., Aközbek N., D’Aguanno G. (2013). Impedance
Matched Thin Metamaterials Make Metals Absorbing. Sci. Rep..

[55] Hwang U., Kim J., Seol M., Lee B., Park I.-K., Suhr J., Nam J.-D. (2022). Quantitative Interpretation
of Electromagnetic Interference
Shielding Efficiency: Is It Really a Wave Absorber or a Reflector?. ACS Omega.

[56] Woltersdorff W. (1934). On the optical
constants of thin metal layers in long-wave ultra-red. J. Phys..

[57] Li S., Xu S., Pan K., Du J., Qiu J. (2022). Ultra-Thin Broadband
Terahertz Absorption and Electromagnetic Shielding Properties of MXene/rGO
Composite Film. Carbon.

[58] Luhmann N., Høj D., Piller M., Kähler H., Chien M.-H., West R. G., Andersen U. L., Schmid S. (2020). Ultrathin
2 Nm Gold as Impedance-Matched Absorber for Infrared Light. Nat. Commun..

[59] Liu Y., Fan R.-H., Qi D.-X., Peng R., Lai Y., Wang M., Luo J. (2026). Breaking the Intrinsic Absorption
Limit for Arbitrarily Thin Conductive Films at Grazing Incidence. Phys. Rev. Lett..

[60] Lee C., Park S. M., Kim S., Choi Y.-S., Park G., Kang Y. C., Koo C. M., Kim S. J., Yoon D. K. (2022). Field-Induced
Orientational Switching Produces Vertically Aligned Ti_3_C_2_T_
*x*
_ MXene Nanosheets. Nat. Commun..

[61] Tabassum S., Dong L., Kumar R. (2018). Determination of Dynamic Variations
in the Optical Properties of Graphene Oxide in Response to Gas Exposure
Based on Thin-Film Interference. Opt. Express.

[62] Jung I., Vaupel M., Pelton M., Piner R., Dikin D. A., Stankovich S., An J., Ruoff R. S. (2008). Characterization
of Thermally Reduced Graphene Oxide by Imaging Ellipsometry. J. Phys. Chem. C.

[63] Downes M., Shuck C. E., McBride B., Busa J., Gogotsi Y. (2024). Comprehensive
Synthesis of Ti_3_C_2_T_x_ from MAX Phase
to MXene. Nat. Protoc..

[64] Roslyk I., Baginskiy I., Zahorodna V., Gogotsi O., Ippolito S., Gogotsi Y. (2024). Porous Ti_3_AlC_2_ MAX Phase Enables
Efficient Synthesis of Ti_3_C_2_T*
_x_
*MXene. Int. J. Appl. Ceram. Technol..

[65] Naguib M., Mochalin V. N., Barsoum M. W., Gogotsi Y. (2014). 25th Anniversary Article:
MXenes: A New Family of Two-Dimensional Materials. Adv. Mater..

[66] Mathis T. S., Maleski K., Goad A., Sarycheva A., Anayee M., Foucher A. C., Hantanasirisakul K., Shuck C. E., Stach E. A., Gogotsi Y. (2021). Modified MAX Phase
Synthesis for Environmentally Stable and Highly Conductive Ti_3_C_2_ MXene. ACS Nano.

[67] Orend K., Baer C., Musch T. (2022). A Compact Measurement Setup for Material
Characterization in W-Band Based on Dielectric Waveguides. Sensors.

[68] Peng M., Qin F. (2021). Clarification of Basic
Concepts for Electromagnetic Interference
Shielding Effectiveness. J. Appl. Phys..

