# Supplementary material for: Anomalous Microwave Absorption Hysteresis in Ultrathin MXene Films with Confined Water
Source: ACS Nano. 2026 Sep 24;20(39):26511–20. doi: 10.1021/acsnano.6c07970 (PMC13648262; doi:10.1021/acsnano.6c07970)
Supplement: Supplementary file 1 [file nn6c07970_si_001.pdf]

Supporting Information

Anomalous Microwave Absorption Hysteresis in Ultrathin MXene Films with Confined Water

Changhoon Park[1],† Jamal AlHourani[1,2],† Yuan Zhang[1], Jongyoun Kim[1], James FitzPatrick[1], Teng Zhang[1], Gary Friedman[2], and Yury Gogotsi[1]**

†These authors contributed equally: Changhoon Park, Jamal AlHourani

[1]Materials Science and Engineering Department and A. J. Drexel Nanomaterials Institute, Drexel University, 3141 Chestnut Street, Philadelphia, PA 19104, USA

[2]Electrical and Computer Engineering Department, Drexel University, 3141 Chestnut Street, Philadelphia, PA 19104, USA

***Corresponding authors:** gogotsi@drexel.edu, gf29@drexel.edu

KEYWORDS MXenes, intercalation, water confinement, electromagnetic interference shielding

EMI measurement experiment

To perform in situ EMI measurements under varying humidity conditions, two WR-90 rectangular waveguides were placed inside a Memmert HCP50 humidity chamber, as shown in Figure S1a. The coaxial cables were connected to the vector network analyzer (VNA) through the opening from the back of the chamber. We calibrated the waveguides using the X11644A Mechanical Calibration Kit at each humidity level to ensure stable, accurate measurements. The MXene films were inserted between the waveguides, as shown in Figure S1b, and the waveguides were aligned and secured to prevent energy leakage. Figure S1c shows the VNA connected to the waveguide via coaxial cables routed through the humidity chamber.

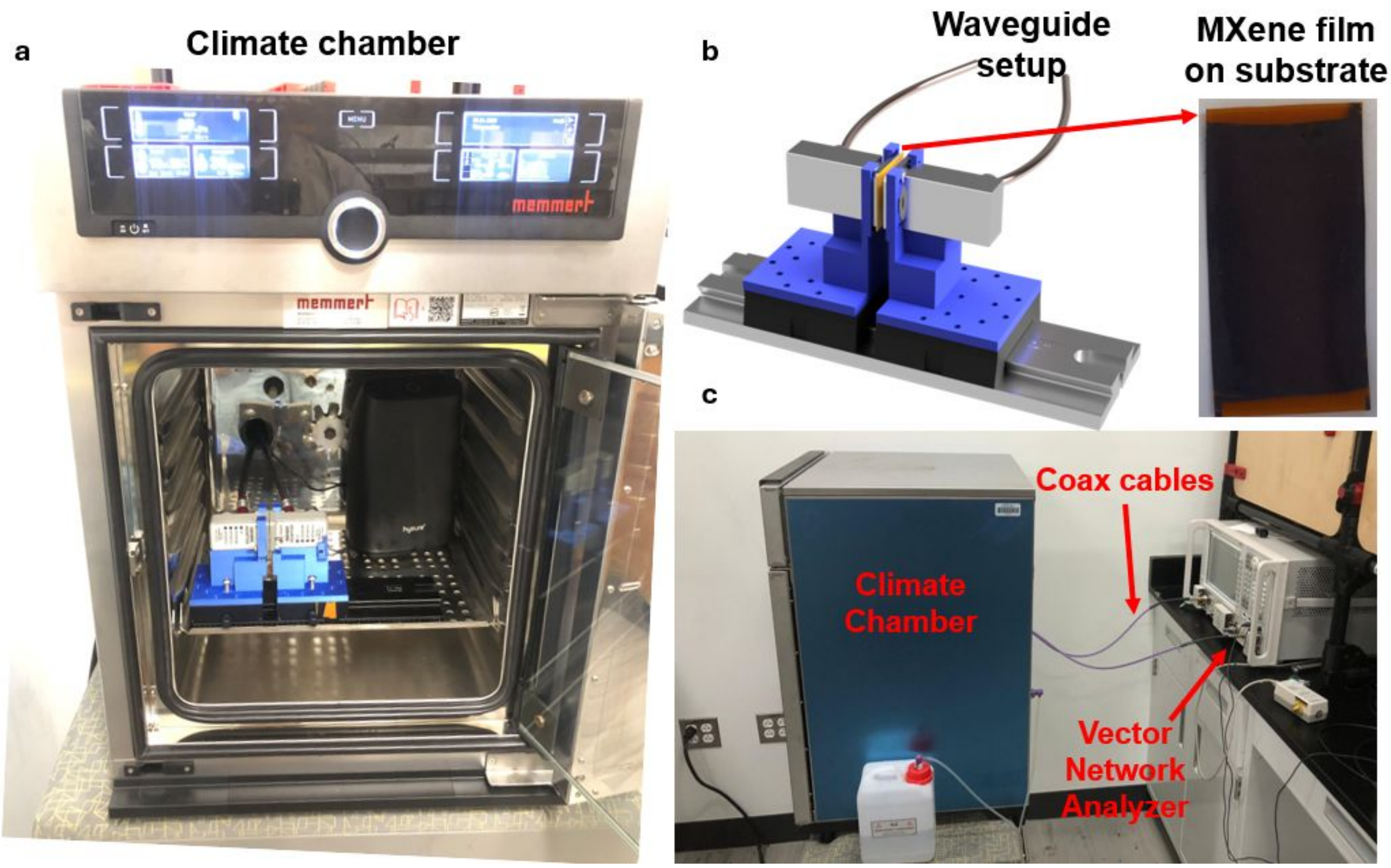

Figure S1. Microwave measurement experimental setup. a) Picture of the rectangular waveguides system inserted inside the climate chamber for the in-situ EMI measurement. b) The two rectangular waveguides with MXene film on a 125 μm polyimide substrate inserted between the waveguide flanges, mounted on an optical rail. c) Picture showing coaxial cables connected from the Vector Network Analyzer to the waveguides inside the chamber.

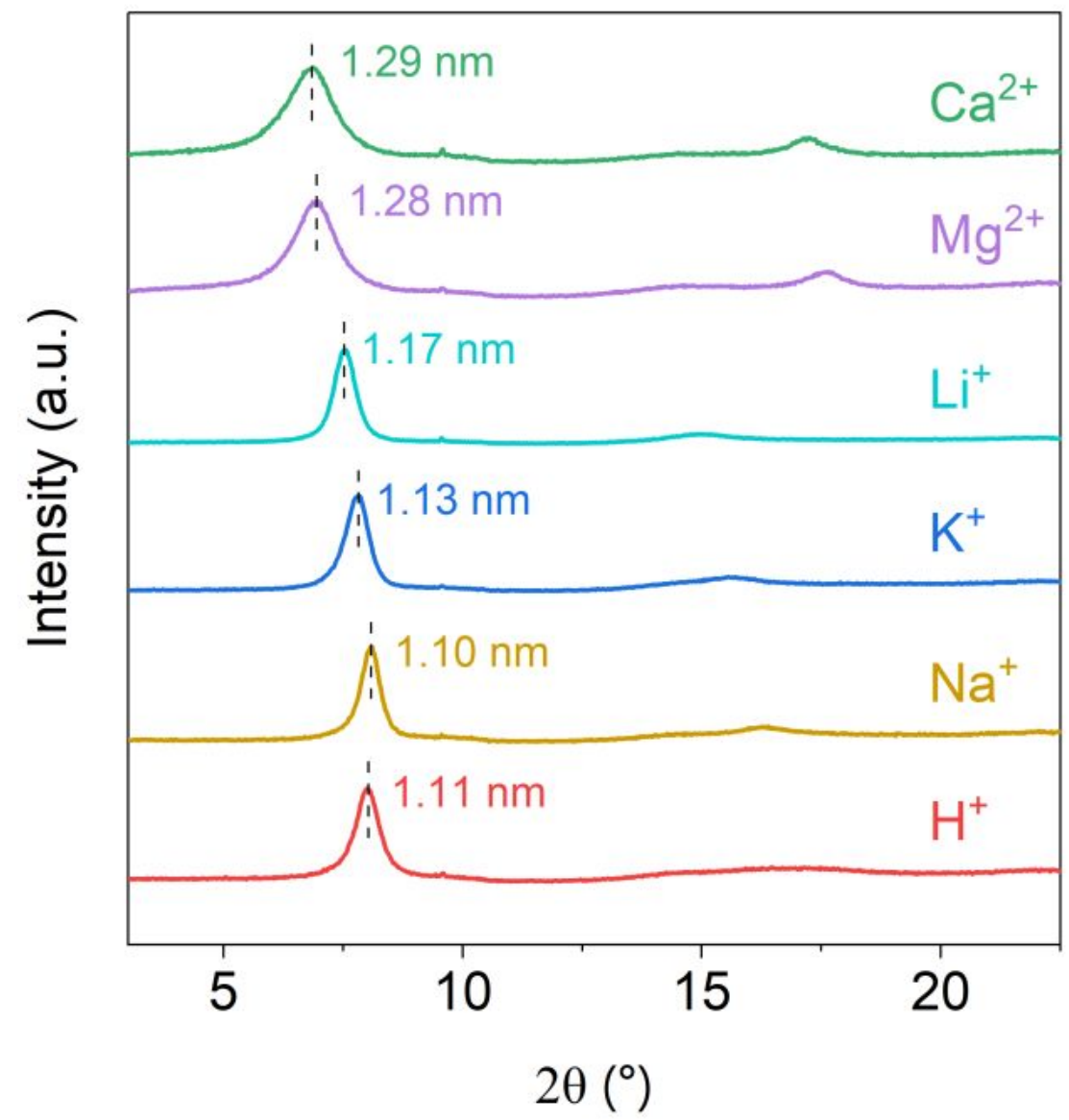

Figure S2. XRD patterns of Ca^{2+}, Mg^{2+}, Li^{+}, K^{+}, Na^{+}, and H^{+}-intercalated MXene films at room temperature and humidity. The shift in the 2θ peaks exhibit the change in the *d*-spacing depending on the type of intercalant used. The films intercalated with larger cations ($Mg^{2+}$and Ca^{2+}) show larger *d*-spacing.

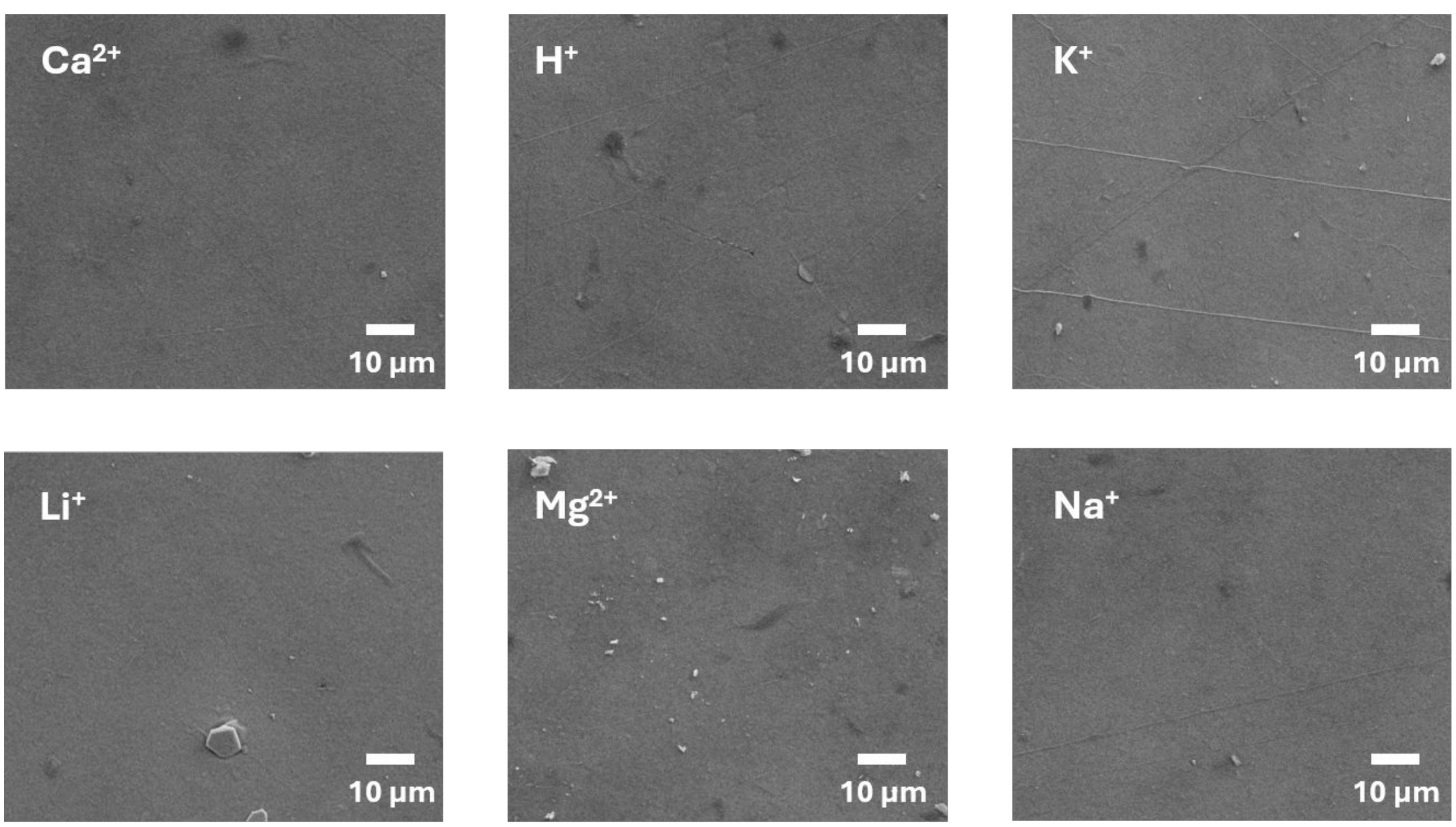

Figure S3. SEM image of the surface of $Ti_3C_2T_x$ MXene films intercalated with Ca^{2+}, H^{+}, K^{+}, Li^{+}, Mg^{2+}, and Na^{+}, respectively.

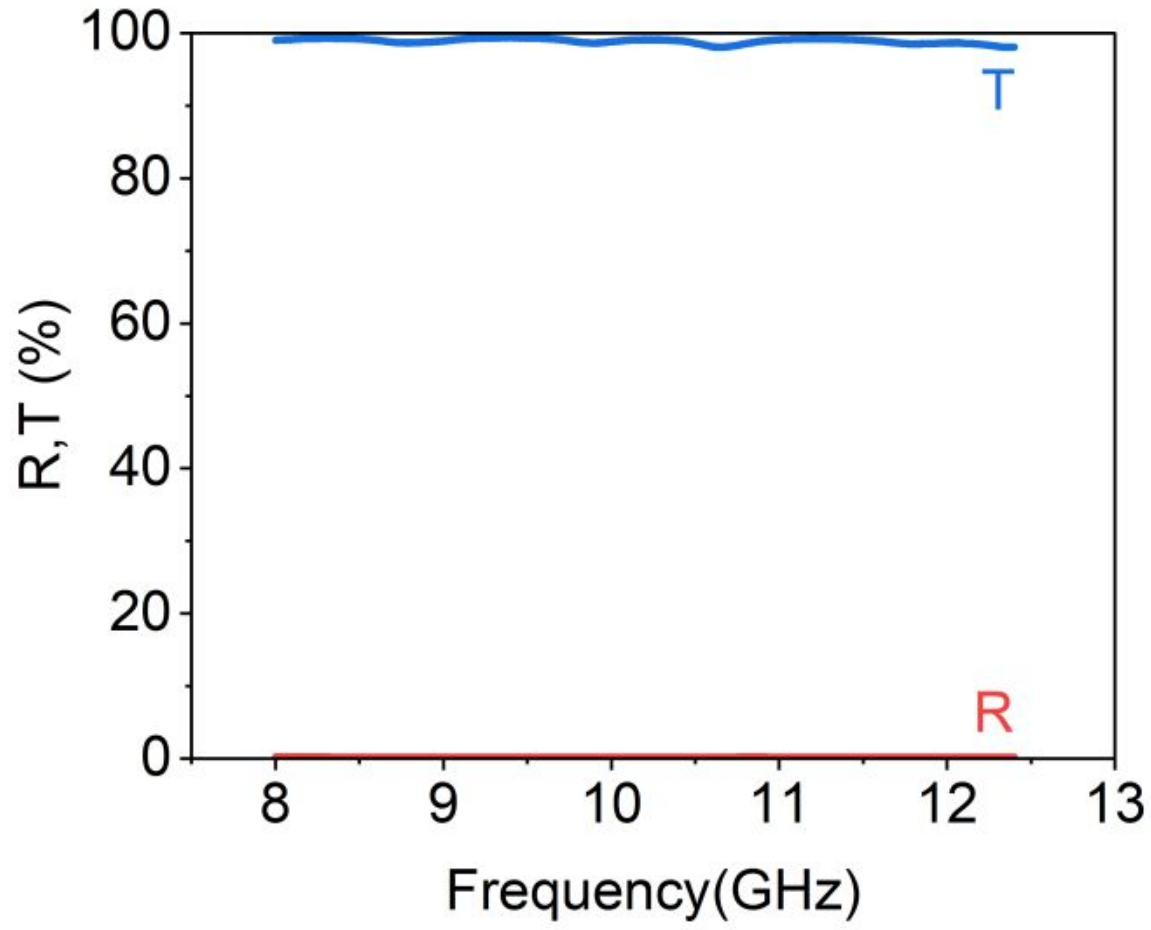

Figure S4. Reflection (R) and transmission (T) spectra as a function of frequency for a 125 µm polyimide substrate, exhibiting transparent EMI properties in the X-band.

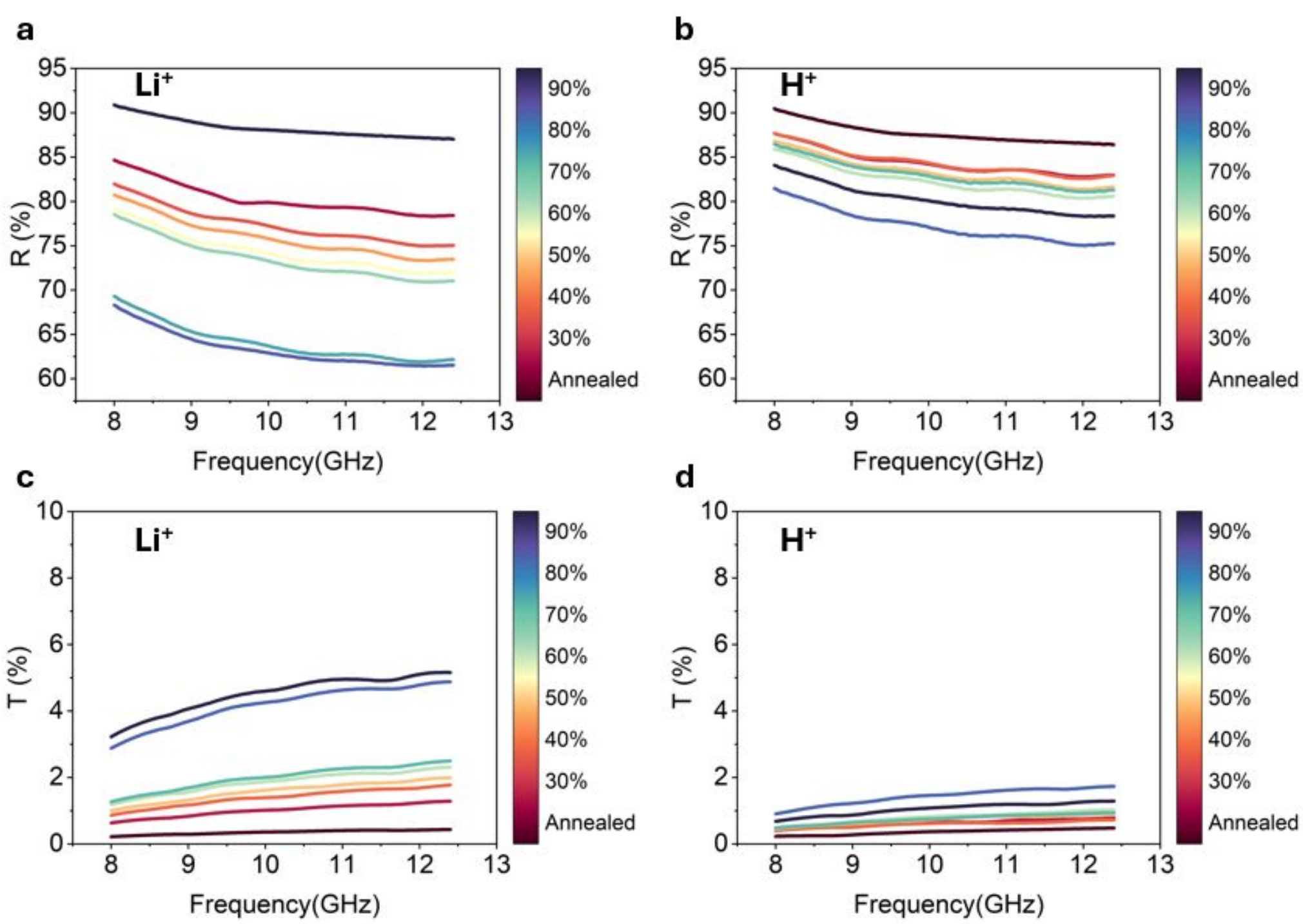

Figure S5. Reflection (R) spectra as a function of frequency for a) Li^+-intercalated, b) H^+-intercalated MXene films. Transmission (T) spectra as a function of frequency for c) Li^+-intercalated, and d) H^+-intercalated MXene films, respectively, across different humidities.

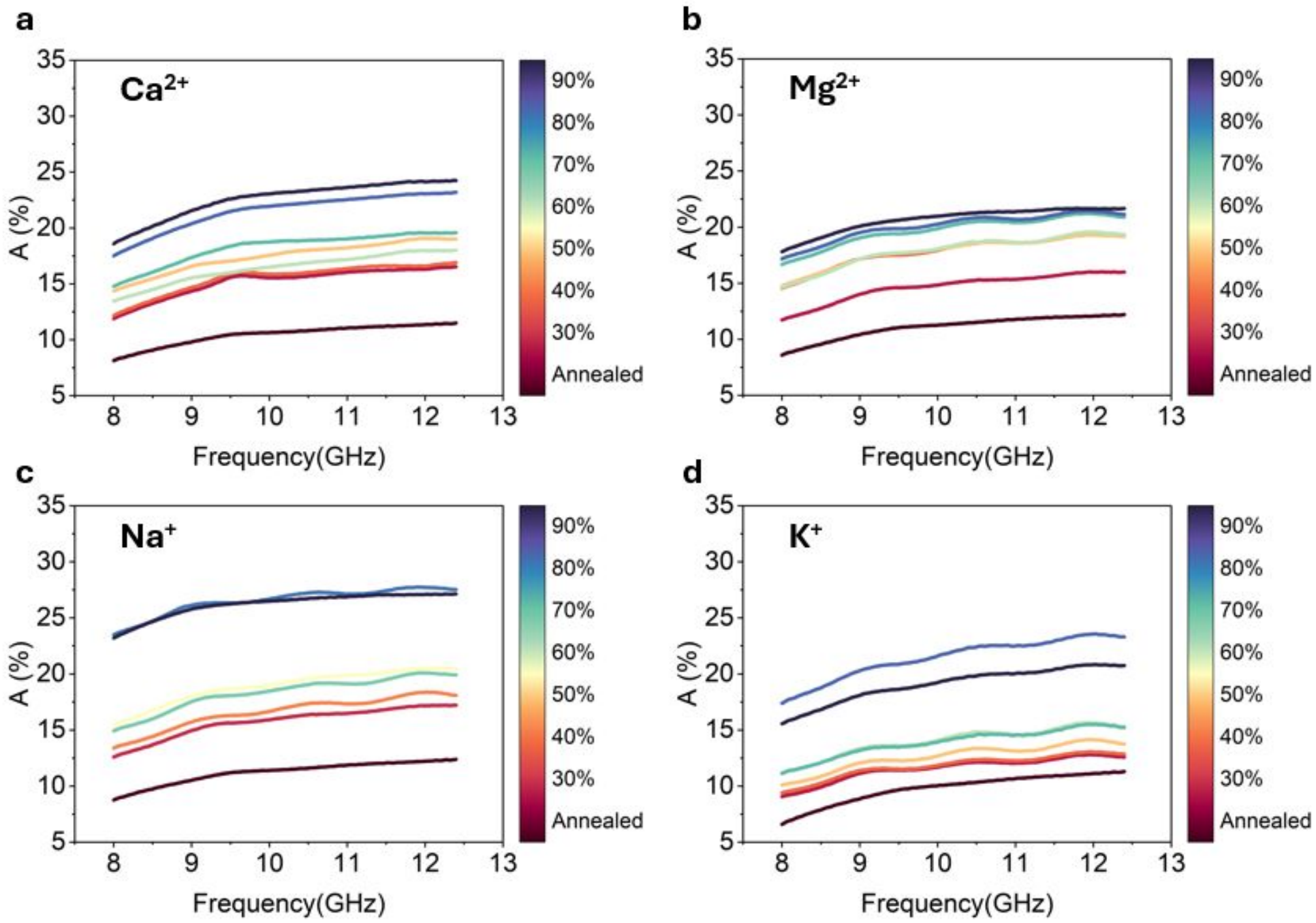

Figure S6. Absorption spectra as a function of frequency for a) Ca^{2+}-intercalated, b) Mg^{2+}-intercalated, c) Na^{+}-intercalated, and d) K^{+}-intercalated MXene films, respectively, across different humidities.

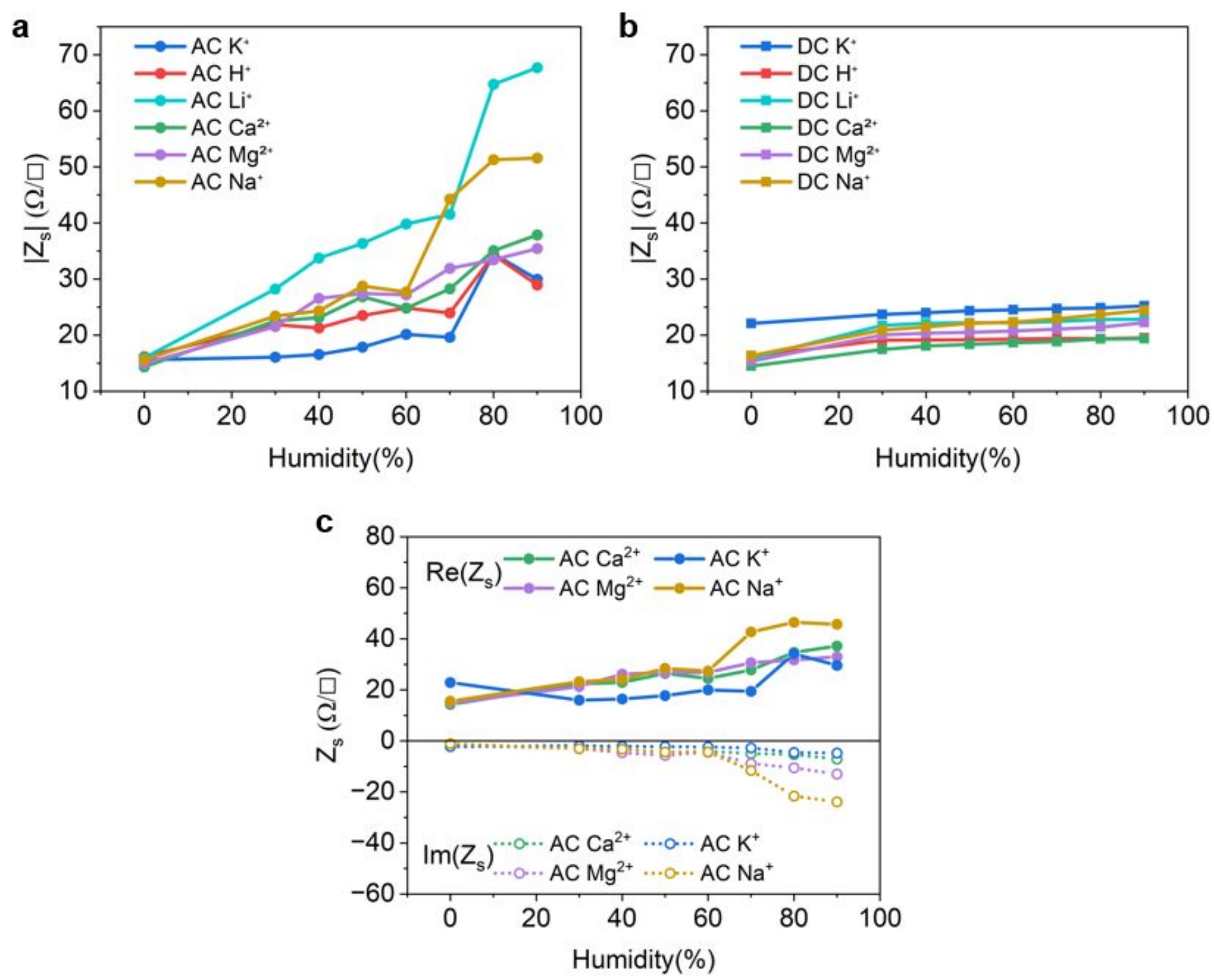

Figure S7. DC and AC sheet impedance behavior of the films. a) Extracted AC sheet impedance magnitude of Li^+, Na^+, Ca^{2+}, Mg, H^+, and K^+-intercalated MXene films as a function of humidity. b) a) Measured DC sheet impedance magnitude of Li^+, Na^+, Ca^{2+}, Mg, H^+, and K^+-intercalated MXene films as a function of humidity. c) Real and imaginary components of the AC sheet impedance of Na^+, Ca^{2+}, Mg, and K^+- intercalated MXene films as a function of humidity.

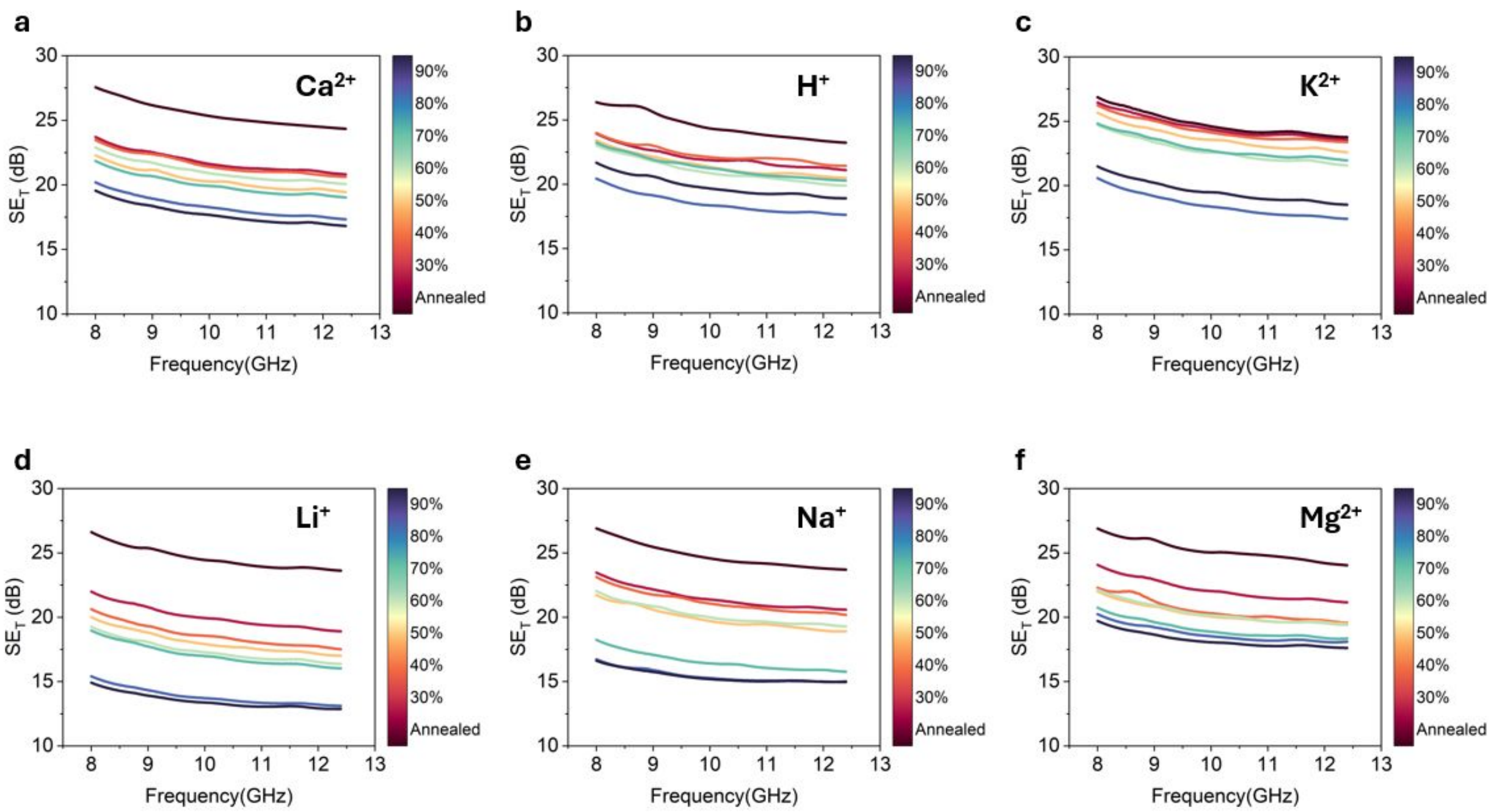

Figure S8. EMI total shielding effectiveness (SE_T) in dB as a function of frequency for a) Ca^{2+}-intercalated, b) H^+-intercalated, c) K^+-intercalated, d) Li^+-intercalated, e) Na^+-intercalated, and f) Mg^{2+}-intercalated films, respectively, across different humidities.

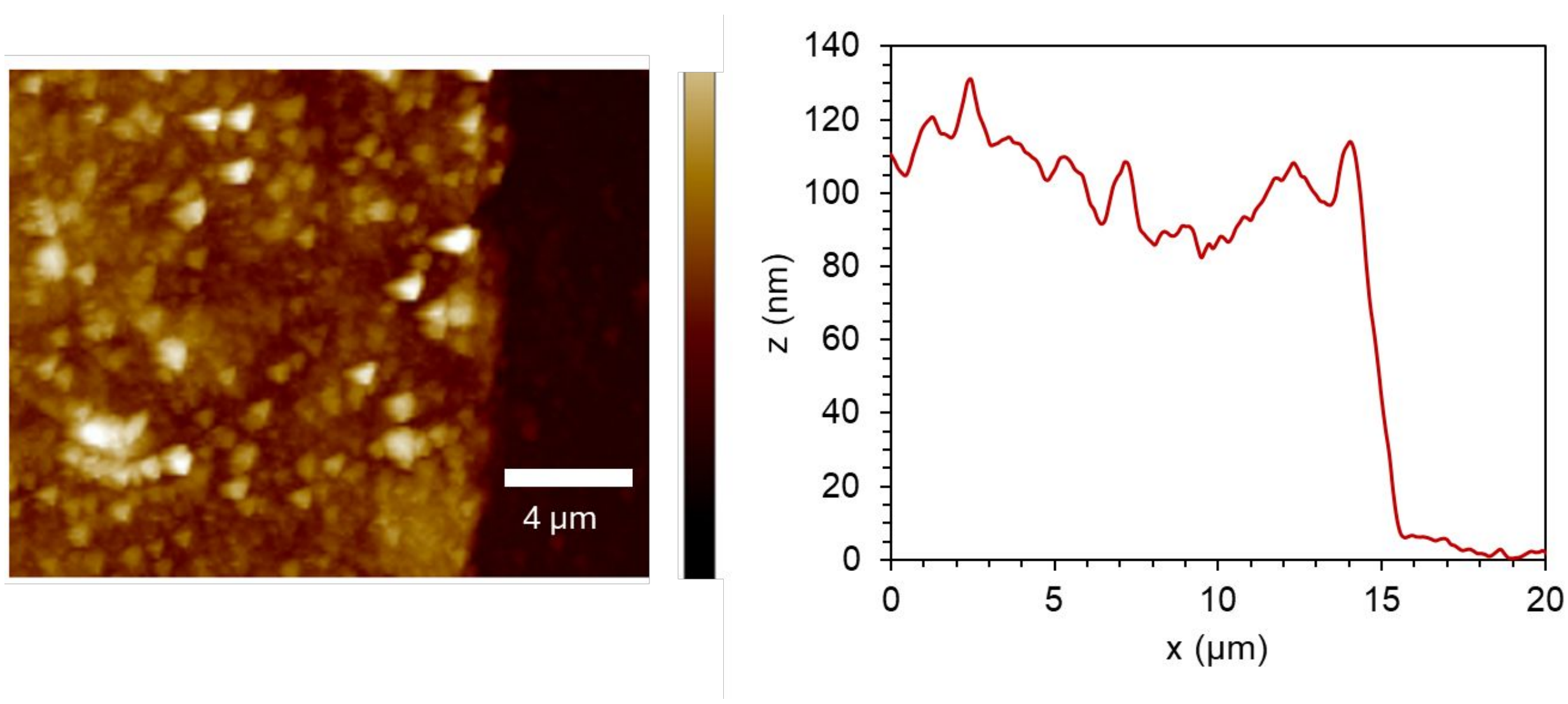

Figure S9. AFM image and a line profile of Li^+-intercalated $Ti_3C_2T_x$ MXene film

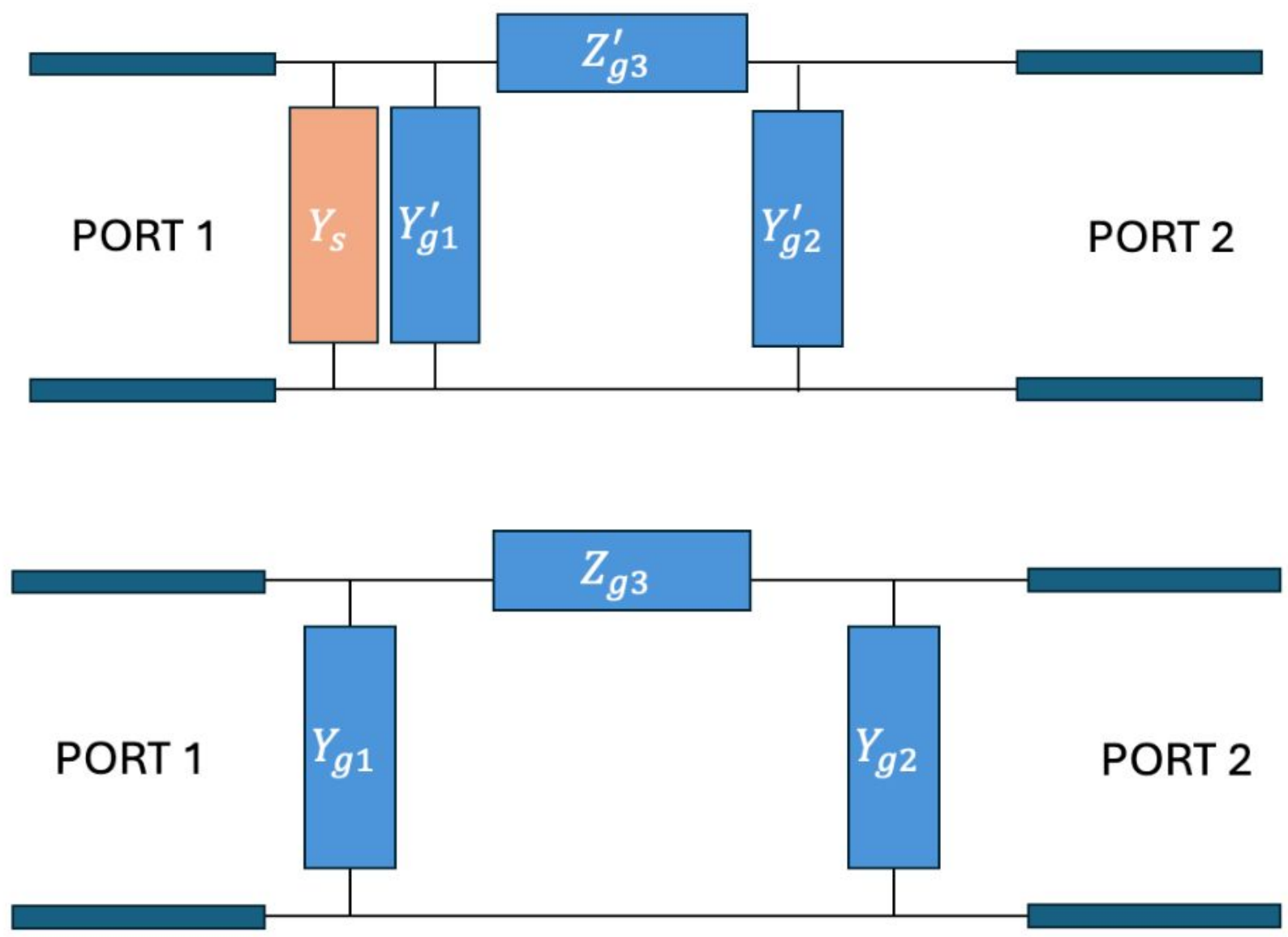

Figure S10. Equivalent transmission line model of the Π-network for (a) bare substrate and (b) substrate with film of sheet resistance

ICP-OES measurement

Elemental analysis was performed using an Agilent 5900 inductively coupled plasma-optical emission spectrometer (ICP-OES). Calibration curves for Li, Na, K, Mg, and Ca were established using standard solutions at concentrations of 0, 0.1, 0.5, 5, and 10 ppm, prepared by serial dilution of certified reference standards (Agilent Technologies) in Milli-Q water. Free-standing films (2-5 mg) following ion exchange were digested in aqua regia at room temperature for 12 h, after which the resulting solutions were collected and diluted tenfold prior to analysis. Measurements were carried out at an RF power of 1.4 kW, with plasma, auxiliary, and nebulizer gas flow rates of 12, 1, and 0.7 L min^{-1}, respectively. Yttrium (Y, 371.029 nm) was used as the internal standard for signal correction and instrumental drift compensation.

	Ca (ppm)	K (ppm)	Li (ppm)	Mg (ppm)	Na (ppm)	Ti (ppm)
H	0.05	n.d.	0.01	n.d.	0.03	28.26
Na	n.d.	0.01	0.01	n.d.	**0.16**	17.67
K	n.d.	**0.91**	n.d.	n.d.	0.05	19.28
Mg	0.01	n.d.	n.d.	**0.27**	0.03	27.28
Ca	**0.49**	n.d.	n.d.	n.d.	0.01	18.91

Table S1. Elemental analysis of $Ti_3C_2T_x$ films after ion-exchange after 10 times of dilution performed using inductively coupled plasma–optical emission spectrometry (ICP–OES).
